# Interfacing with silica boosts the catalysis of copper

**DOI:** 10.1038/s41467-018-05757-6

**Published:** 2018-08-22

**Authors:** Chaofa Xu, Guangxu Chen, Yun Zhao, Pengxin Liu, Xinping Duan, Lin Gu, Gang Fu, Youzhu Yuan, Nanfeng Zheng

**Affiliations:** 1 State Key Laboratory for Physical Chemistry of Solid Surfaces, Collaborative Innovation Center of Chemistry for Energy Materials, National & Local Joint Engineering Research Center for Preparation Technology of Nanomaterials, and National Engineering Laboratory for Green Chemical Productions of Alcohols–Ethers–Esters, College of Chemistry and Chemical Engineering, Xiamen University, Xiamen, 361005 China; 2 Institute of Physics, Chinese Academy of Sciences, Beijing, 100190 China

## Abstract

Metal-support interaction is one of the most important parameters in controlling the catalysis of supported metal catalysts. Silica, a widely used oxide support, has been rarely reported as an effective support to create active metal-support interfaces for promoting catalysis. In this work, by coating Cu microparticles with mesoporous SiO_2_, we discover that Cu/SiO_2_ interface creates an exceptional effect to promote catalytic hydrogenation of esters. Both computational and experimental studies reveal that Cu–H^δ−^ and SiO–H^δ+^ species would be formed at the Cu–O–SiO_*x*_ interface upon H_2_ dissociation, thus promoting the ester hydrogenation by stablizing the transition states. Based on the proposed catalytic mechanism, encapsulting copper phyllosilicate nanotubes with mesoporous silica followed by hydrogen reduction is developed as an effective method to create a practical Cu nanocatalyst with abundant Cu-O-SiO_*x*_ interfaces. The catalyst exhibits the best performance in the hydrogenation of dimethyl oxalate to ethylene glycol among all reported Cu catalysts.

## Abstract (web summary)

Metal-support interaction plays an important role in heterogeneous catalysis, but silica has been rarely reported as an effective support to create active metal-support interfaces for promoting catalytic reactions. Here, the authors discover that Cu/SiO_2_ interface creates an exceptional effect to promote catalytic hydrogenation of esters.

## Introduction

Heterogeneous catalysis is of vital importance in many fields of chemical, food, energy, and environmental applications. The rational design and fabrication of sufficient active interfaces between metal and (hydr)oxide to facilitate the reactions with multiple reagents has emerged as an effective strategy to prepare heterogeneous catalysts with improved performances. For instance, both Pt/FeO_*x*_ and Pt/Fe(OH)_*x*_ interfaces exhibit excellent performance in CO oxidation and CO preferential oxidation (PROX)^1–4^. Au/CeO_*x*_ and Au/TiO_*x*_ interfaces have been demonstrated to improve the activity of water-gas shift reaction^5–7^. Pt/M(OH)_2_ (M = metal) interfaces enhance the performance of hydrogen evolution reaction and hydrogen oxidation reaction^8–11^. Such interfacial effects from the strong metal–metal (hydr)oxide interactions were typically observed only when reducible metal oxides (e.g., TiO_*x*_, CeO_*x*_, FeO_*x*_) were used as supports^12–17^. In contrast, SiO_2_ without reducible metal cations usually serves as ‘inert’ support, or plays as shell material to fabricate yolk-shell and core-shell metal nanocatalysts to prevent the sintering of metal components^18–24^. Reports on the promotional effects of SiO_2_ on heterogeneous catalysis are rare^25,26^.

Here we demonstrate that SiO_2_ readily creates highly active interfaces with Cu in the gas-phase hydrogenation of dimethyl oxalate (DMO) into ethylene glycol (EG). In this work, the Cu-SiO_2_ interfaces were first designed and fabricated by depositing a mesoporous SiO_2_ (m-SiO_2_) layer onto the surface of commercial Cu powders. With the created Cu–SiO_2_ interfaces, the coated Cu powders exhibited a two-order-of-magnitude enhancement in the activity as compared to the uncoated Cu powders. Combining experiments with density functional theory (DFT) calculations, we demonstrate that H_2_ could be activated at the Cu^δ+^–O–SiO_*x*_ interface region, giving rise to Cu–H and interfacial SiO–H species, which are able to promote the hydrogenation of polar C=O bonds. Based on this understanding, a smart strategy by in situ reducing silica coated copper phyllosilicate nanotubes was developed to produce a sophisticated Cu-SiO_2_ nanocatalyst with abundant Cu–O–SiO_*x*_ interface. Such a catalyst exhibited the best reported performance in selective hydrogenation of DMO to EG.

## Results and Discussion

### Cu–O–SiO_*x*_ interfaces boost the catalysis of copper

**Fig. 1:**
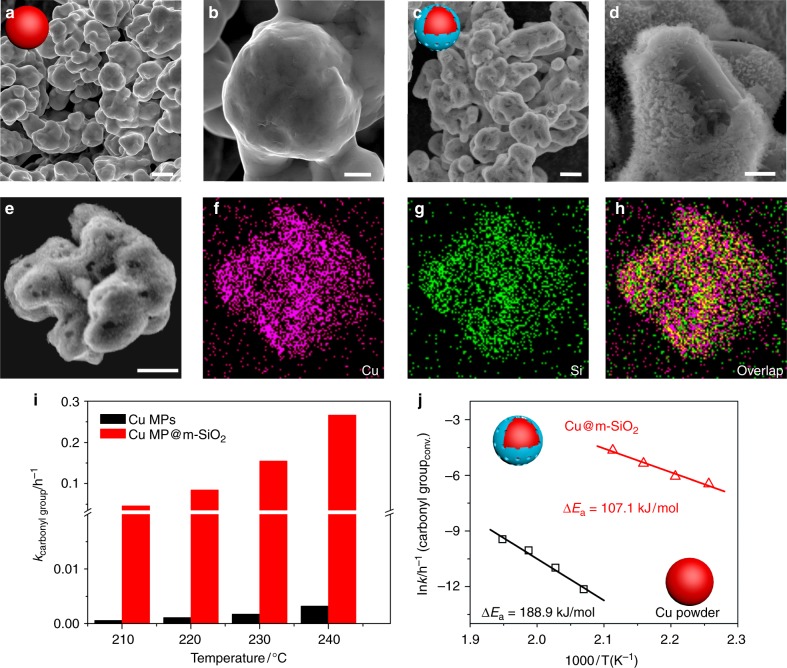
Demonstration of Cu–O–SiO_*X*_ interface effect in DMO hydrogenation. **a**–**d** SEM images of Cu microparticle before (**a**, **b**) and after (**c**, **d**) coating mesoporous silica; **e**–**h** EDX mapping images of mesoporous silica coated Cu microparticles (Cu@m-SiO_2_); **i**, **j** Catalytic performance and the apparent activation energy (*E*_a_) of Cu microparticles before and after coating mesoporous silica for the selective hydrogenation of DMO, respectively; Reaction conditions were as follows: H_2_/DMO = 80 mol/mol, *P* (H_2_) = 3.0 MPa. Scale bars in **a**, **c** and **e** are 2 µm. Scale bars in **b** and **d** are 500 nm

To create Cu–O–SiO_*x*_ interfaces, a non-continuous layer of m-SiO_2_ was deposited onto the surface of commercial Cu microparticles (MPs) with diameter of 2–3 µm by hydrolysis of tetraethoxysilane (TEOS) in the presence of cetyltrimethylammonium bromide (CTAB) (See 'Methods' section). Scanning electron microscopy (SEM) and energy dispersive spectroscopy (EDS) analysis (Fig. 1a–h, Supplementary Fig. 1) revealed the successful deposition of a downy layer of SiO_2_ on Cu MPs. The mesoporous nature of the SiO_2_ layer deposited on Cu MPs was confirmed by the N_2_ adsorption and desorption isotherm at 77 K (Supplementary Fig. 2). It should also be noted that the m-SiO_2_ layer was not continuously grown on Cu, resulting in the exposure of partial Cu sites on the as-obtained hybrid of Cu-MP@m-SiO_2_.

To evaluate the Cu–O–SiO_*x*_ interfacial effect, we chose the gas-phase hydrogenation of DMO.^27–29^ As shown in Fig. 1i, Cu MPs without SiO_2_ coating displayed a negligible activity in the hydrogenation of DMO at the temperature below 250 °C. In comparison, Cu MPs coated with m-SiO_2_ exhibited a significant activity even at the temperature of 210 °C. The turnover rate of carbonyl groups (*k*_carbonyl group_) over Cu-MP@m-SiO_2_ was approximately 80 times higher than that on uncoated Cu MPs at the temperature between 200 and 240 °C. In this comparison, the *k*_carbonyl group_ was calculated based on the hydrogenation rate of carbonyl groups over the total amount of Cu in the catalysts. The hydrogenation activity of the Cu-MP@m-SiO_2_ catalyst was increased with the temperature. Considering that less Cu sites were exposed on Cu-MP@m-SiO_2_, the catalytic enhancement induced by the Cu–O–SiO_*x*_ interfaces was tremendous. Moreover, the apparent activation energy (*E*_a_) over Cu-MP@m-SiO_2_ was measured to be 107.1 kJ mol^−1^ (Fig. 1j), almost only half of that on Cu MPs (188.9 kJ mol^−1^), indicating the as-built Cu-O-SiO_*x*_ interfaces would completely alter the hydrogenation mechanism.

### Hydrogenation mechanism over the Cu–O–SiO_*x*_ interface

The promotional effect of the Cu–O–SiO_*x*_ interface on the catalytic hydrogenation of DMO was studied by using DFT calculations. In this work, structural models of periodic Cu(111) with/without SiO_2_ coating were built to simulate the modified and unmodified Cu MPs, respectively. Until now, it is still a great challenge to identify the interfacial structure between metal and silica since the SiO_2_ deposition could present as various crystalline or vitreous films. To simplify the interface model, we assumed that the [SiO_4_] tetrahedra could be stacked on Cu(111) in a two-dimensional ordered network with a composition of SiO_2.5_, in which every Si has one Si–O–Cu bond and three Si–O–Si bonds (Supplementary Fig. 3). According to our DFT calculations, the proposed model was calculated to be exothermic by 0.66 eV/Si with respective to Cu(111), α-quartz SiO_2_ and gaseous O_2_. Bader charge analysis showed that surface Cu atoms, which were directly bonded with O-SiO_3_, would carry significantly positive charge, implying that the SiO_2_ overgrowth would lead to the formation of Cu^δ+^ species (Supplementary Fig. 3). Interestingly, similar silica films with c(2 × 2) structures have been demonstrated to form on Mo(112) and Ru(0001) single crystal surfaces^30–32^. As suggested previously, the adsorption energy of oxygen atoms plays an important role in determining whether silica monolayer film can be stabilized on the metal surface or not. In our case, the dissociative adsorption energy of O_2_ on Cu(111) was calculated as −3.13 eV, just between those of Mo(112) (−5.64 eV) and Ru(0001) (−2.28 eV). In addition, Cu(111) has lattice constant of 2.556 Å, matching well with the silica film with a 5.2~5.3 Å periodicity in the (2 × 2) manner. All these results indicated that the formation of monolayer SiO_2_ network over Cu(111) surfaces is reasonable. On the surface of Cu-MP@m-SiO_2_, there were still a large amount of uncovered Cu atoms so that the silica film should not be continuous. To account for the experimental observation, we extended our model to a (8 × 4) structure in which 50% [SiO_4_] tetrahedra were removed, and the as-generated Si–O dangling bonds were saturated by H atoms. Thus, the Cu–O–SiO_*x*_ interface turned to be accessible for the substrate molecules. Hereafter, such a model is denoted as SiO_2_/Cu(111).

Hydrogenation on the heterogeneous catalysts usually follows Horiuti Polanyi mechanism, which consists of following steps: (i) dissociation of hydrogen; (ii) adsorption of unsaturated compounds; (iii) stepwise hydrogenation with H atoms. Supplementary Fig. 4 showed the dissociation of H_2_ on the two distinct surfaces, i.e., Cu(111) and SiO_2_/Cu(111). On Cu(111), H–H bond splitting occurred via a homolytic mechanism, generating two hydrogen atoms adsorbed on the three-fold sites. Alternatively, the presence of Cu–O–SiO_*x*_ interface on SiO_2_/Cu(111) enable H_2_ activation in a heterolytic way, yielding Cu–H and interfacial SiO–H species simultaneously at the interface. From Cu(111) to SiO_2_/Cu(111), the calculated barrier for H_2_ dissociation does not change too much (0.28 eV vs. 0.30 eV), indicating that heterolytic dissociation could be competitive with the hemolytic one. However, from the viewpoint of thermodynamics, H_2_ dissociation on SiO_2_/Cu(111) was found to be more exothermic than that on Cu(111) (−0.81 eV vs. −0.47 eV) because the interfacial SiO–H bond is stronger than Cu–H bond. Therefore, even the H_2_ dissociation occurred via the hemolytic route, interfacial SiO–H^δ+^ species would be generated through thermodynamics-driven hydrogen spillover. All these indicated that there existed abundant interfacial SiO–H^δ+^ and Cu–H^δ−^ at the Cu–O–SiO_*x*_ interfaces upon hydrogenation.

**Fig. 2:**
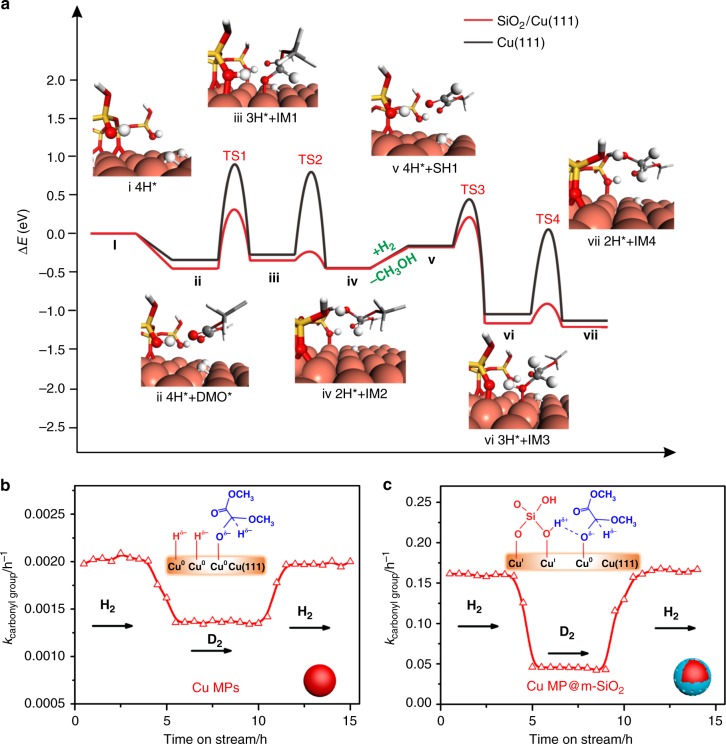
Mechanism of DMO hydrogenation on Cu–O–SiO_*x*_ interface. **a** The DMO hydrogenation pathway on Cu(111) and SiO_2_/Cu(111); **b**, **c** The kinetic isotope effect of Cu MPs catalyst and Cu MPs@m-SiO_2_ catalyst in hydrogenation of DMO. Reaction conditions were as follows: H_2_/DMO = 80 mol/mol, *P* (H_2_) = 3.0 MPa, *T* = 230 °C

The DMO hydrogenation pathways on Cu(111) and SiO_2_/Cu(111) were compared, with the optimized structures of transition states (TS’) and important intermediates (IMs) illustrated in Fig. 2a, Supplementary Figs. 5 and 6. For clarity, only the lowest energy pathways were shown for these two surfaces. According to our DFT calculations, DMO was weakly adsorbed on both Cu(111) and SiO_2_/Cu(111) (from i to ii in Fig. 2a). In this regard, there are two possible mechanisms, namely hydroxyl mechanism and alkoxy mechanism. In the former case, the first hydrogen atom would attack the O end of C=O group, producing the hydroxyl intermediate, while in the latter case, C end of C=O group would be hydrogenated first, leading to the formation of alkoxy intermediate. It has been reported previously that hydroxyl intermediate was thermodynamically less favorable than alkoxy intermediate^33,34^. On both surfaces, the hydrogenation of DMO begins with the nucleophilic attack of H^δ−^ to the electron deficient carbon of ester group. On Cu(111), a 1.22 eV barrier (TS1) has to be surmounted when the initial hydrogenation takes place. In contrast, a relative low barrier of 0.77 eV (TS1) is required when the reaction occur at the Cu–O–SiO_*x*_ interface. It should be noted that both of the reactions proceed with similar endothermicity of ~0.10 eV, indicating that the difference in the barrier should be attributed to the electronic effect (from ii to iii in Fig. 2a). Based on the Bader charge analysis, it was found that when H^δ−^ approaches, the adsorbed DMO would bear a ~ −0.5 a.u. charge. Such a negatively charged TS can be stabilized by the SiO–H^δ+^ species, but repulsive with the co-adsorbed Cu–H^δ−^. This finding nicely explained why the addition of ‘inert’ silica could significantly enhance the hydrogenation.

Next, the half-hydrogenated intermediate undergoes the second H addition to give alcohol species (iv in Fig. 2a IM2). On Cu(111), the TS2 occurs through a reductive elimination of CH_3_O(O=)C–CH(OCH_3_)O^δ−^ and H^δ−^, yielding a barrier of 1.07 eV. Such a high barrier might be originated from the electrostatic repulsion between two negatively charged species. On the contrary, SiO-H^δ+^ at the Cu–O–SiO_*x*_ interface can provide a proton, which is quickly transferred to CH_3_O(O=)C–CH(OCH_3_)O^δ−^ to make a O–H bond by passing a small barrier of 0.11 eV (from iii to iv in Fig. 2a). From this result, the interfacial SiO–H^δ+^ species should be very acidic since the Cu atoms at the interface can stabilize their deprotonated forms. The proposed mechanism nicely explains why the Cu–O–SiO_*x*_ interface is a good choice for the hydrogenation reaction. On one hand, the Cu–OSi bond is not so strong to inhibit the dissociation of H_2_. On the other hand, moderate Cu–OSi bond would render interfacial SiO–H bond suitable strength, which not only stabilizes the charged TS’ but also releases a proton when necessary.

For IM2, the co-presence of OH and OMe groups on the same carbon atoms makes them unstable, which can be easily converted into adsorbed CH_3_O(O=)C–CHO (SH1) plus a methanol molecule (from iv to v in Fig. 2a). Subsequently, SH1 would undergo step-wise hydrogenation again, passing through TS3 and TS4, leading to formation of MG species (IM4). Again, the calculated barriers for TS3 and TS4 on SiO_2_/Cu(111) were much lower than those on Cu(111), and the hydrogenation of aldehyde groups was easier than that of the ester groups (from v to vii in Fig. 2a). Similarly, MG can further be hydrogenated into EG by consecutive H atoms addition. DFT calculations show that the hydrogenation of MG is harder to be conquered than that of DMO. Either on SiO_2_/Cu(111) or Cu(111), the energy gaps between TS5 and TS1 are nearly the same (0.42 eV ~0.45 eV) (from viii to ix in Supplementary Fig. 5). Structurally, DMO has two ester groups. When one of them is hydrogenated, the other serves as an electron-withdrawing group which can stabilize charged TS’ through electron delocalization. Unfortunately, the CH_2_OH group in MG lacks the ability to delocalize the negative charge upon ester hydrogenation. Thus, the rate determining step for the hydrogenation of DMO to EG is corresponding to the hydrogenation of MG.

The mechanism suggested by DFT calculations was verified by serial isotope-labeling experiments (Fig. 2b). The kinetic isotope effect (KIE) of Cu MPs@m-SiO_2_ catalyst (*k*_H_/*k*_D = _3.5) is about two times higher than that of Cu MPs catalyst (*k*_H_/*k*_D = _1.5), indicating the different hydrogenation mechanisms on the two catalysts. As shown in Supplementary Fig. 5, not only the Cu–H but also the SiO–H are involved in the TS1 state as well as TS5 on SiO_2_/Cu(111). It was expected that when hydrogen was replaced by deuterium, the vibrations of both Cu–D and SiO–D would contribute to the zero-point energy of TS’, leading to a large KIE. It is particularly interesting that, when Cu MPs@m-SiO_2_ was treated by a NaOH solution, Na^+^ ions pre-occupies the Cu–SiO sites so that the formation of SiO–H should be suppressed during the hydrogenation. As expected, the TOF for the NaOH-treated Cu MPs@m-SiO_2_ catalyst was decreased dramatically to a similar level to that of uncoated Cu MPs, and the KIE was also dropped back to 1.6 (Supplementary Fig. 7). With the combination of DFT calculation and isotope-labelling experiments, we conclude that the Cu–O–SiO_*x*_ interface not only activates H_2_ molecules in the heterolytic way to form Cu–H^δ−^ and SiO–H^δ+^, but also facilitates the hydrogenation of ester by stablizing the transition states.

### Nanostructure engineering enriches the Cu–O–SiO_*x*_ interfaces

**Fig. 3:**
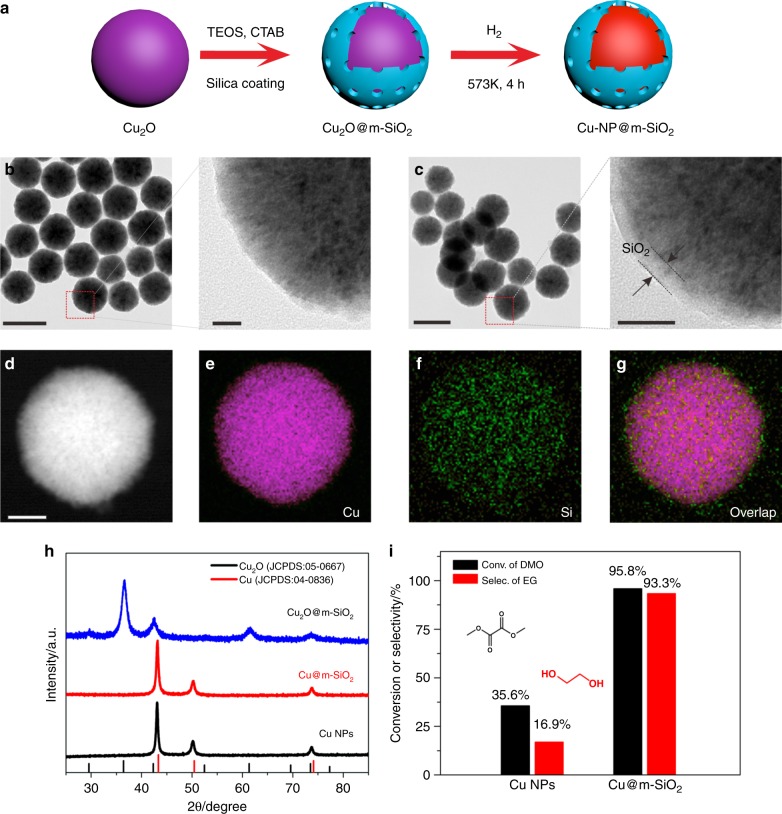
Creating Cu–O–SiO_*x*_ interfaces on Cu nanoparticles. **a** Scheme for the synthesis of Cu@m-SiO_2_; **b**, **c** TEM images of as-prepared Cu_2_O nanoparticles and Cu_2_O@m-SiO_2_, respectively. **d**–**g** EDX mapping images of Cu_2_O@m-SiO_2_; **h** X-ray powder diffraction (XRD) pattern of Cu NPs, Cu_2_O@m-SiO_2_ and Cu@m-SiO_2_; **i** Catalytic performance of Cu NPs and Cu@m-SiO_2_ for the selective hydrogenation of DMO; Reaction conditions were as follows: H_2_/DMO = 80 mol/mol, *P* (H_2_) = 3.0 MPa, *T* = 200 °C, LHSV = 2.4 h^−1^. Scale bars in **b** (left) and **c** (left) are 200 nm. Scale bars in **b** (right) and **c** (right) are 20 nm. Scale bar in **d** is 50 nm

As the promotional effect induced by the Cu–O–SiO_*x*_ interface, increasing Cu–O–SiO_*x*_ interfaces should lead to further enhanced catalytic efficiency. Thus, reducing the size of Cu particles to the nanoscale would amplify the interfacial effect. To create Cu–SiO_2_ interface on Cu nanoparticles, Cu_2_O nanoparticles were first prepared and coated by m-SiO_2_ (Fig. 3a). The obtained Cu_2_O@m-SiO_2_ nanoparticles were then reduced under H_2_ atmosphere to convert into Cu@m-SiO_2_ nanoparticles (denoted as Cu-NP@m-SiO_2_). Comprehensive characterizations by TEM, EDS, XRD (Fig. 3b–h), and N_2_ adsorption/desorption isotherms (Supplementary Fig. 8) confirmed the core-shell structure of Cu-NP@m-SiO_2_. As expected, the as-prepared Cu-NP@m-SiO_2_ catalyst exhibited a much better catalytic performance in DMO hydrogenation than unmodified Cu NPs (Fig. 3i). With a liquid hourly space velocity (LHSV) of 2.4 h^−1^, Cu-NP@m-SiO_2_ exhibited both much higher DMO conversion (95.8%) and higher selectivity (93.3% to EG) at 200 °C. In contrast, when Cu NPs were used as the catalyst, only 35.6% of DMO was hydrogenated with 16.9% of selectivity to EG.

**Fig. 4:**
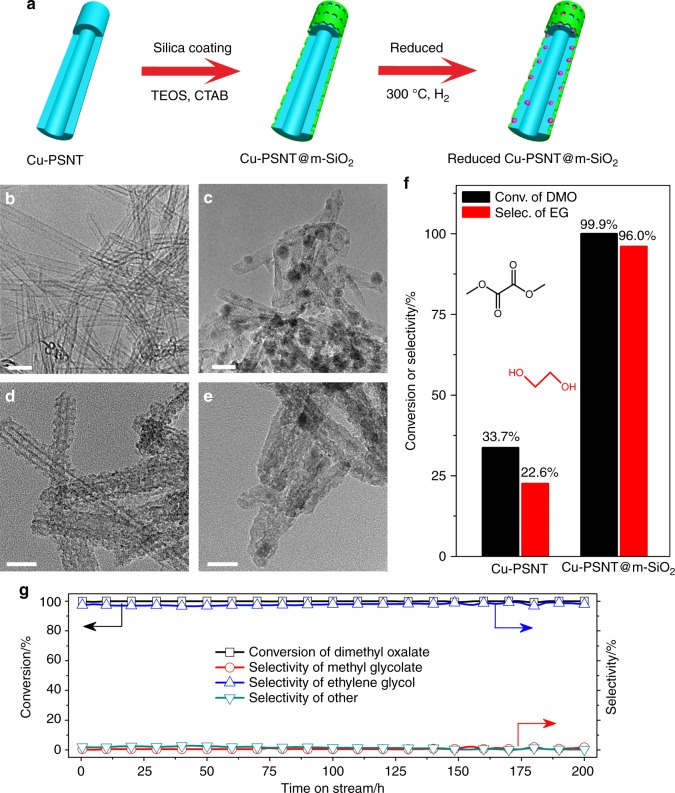
Confined growth strategy for maximizing Cu–O–SiO_*x*_ interface. **a** Illustration of the synthetic strategy for the preparation of Cu-PSNT@m-SiO_2_; **b**–**e** TEM image of as-prepared Cu-PSNT, reduced Cu-PSNT, Cu-PSNT@m-SiO_2_ and reduced Cu-PSNT@m-SiO_2_, respectively; **f** Catalytic performance of reduced Cu-PSNT and reduced Cu-PSNT@ m-SiO_2_ for the selective hydrogenation of DMO to EG (LHSV = 7.8 h^−1^); **g** Catalytic performance of reduced Cu-PSNT@m-SiO_2_ catalyst as a function of time-on-stream (LHSV = 2.0 h^−1^). Reaction conditions were as follows: H_2_/DMO = 80 mol/mol, *P* (H_2_) = 3.0 MPa, *T* = 200 °C. Scale bars are 20 nm for (**b**–**e**)

In term of Cu utilization, the core-shell overgrowth structure (Cu-NP@m-SiO_2_) demonstrated above was not the ideal structure for practical applications because most of Cu atoms in Cu-NP@m-SiO_2_ were not located on surface or Cu–O–SiO_*x*_ interfaces. In this regard, encapsulating ultra-small Cu nanoparticles in a porous SiO_2_ matrix should be the most effective strategy to create highly active catalysts while maximizing the utilization of Cu. For this purpose, we chose copper phyllosilicate nanotubes as an alternative Cu precursor. Structurally, copper phyllosilicate has lamellar structure composed of alternate layers of SiO_4_ tetrahedra and discontinuous layers of CuO_6_ octahedra, in which Cu–O–SiO_*x*_ moieties are readily available (Supplementary Fig. 9)^35–39^. By using a modified hydrothermal method, copper phyllosilicate nanotubes (Cu-PSNT) with sub-10 nm in diameter, 1–2 nm in wall thickness, and hundreds of nanometers in length were prepared (Fig. 4b). Both TEM and EDS analysis (Fig. 4c, Supplementary Fig. 10) confirmed the formation of metallic Cu nanoparticles which were embedded in SiO_2_ matrix after the H_2_ reduction. Some large Cu nanoparticles with size larger than 10 nm were also observed. Moreover, the BET analysis (Supplementary Fig. 11) revealed that the reduced Cu-PSNT had a BET surface area of 470.1 m^2^ g^−1^ and a pore volume of 1.47 cm^3^ g^−1^.

To demonstrate the advantages of the reduced Cu-PSNT in catalysis, a Cu/SiO_2_ catalyst (denoted as Cu/SiO_2_-AE, Supplementary Fig. 12) was prepared by a reported ammonia-evaporation method for comparison^38,40–42^. The Cu/SiO_2_-AE catalyst represents the state-of-the-art Cu catalyst reported in the literature for the selective hydrogenation of DMO to EG^29,43–45^. Although Cu nanoparticles in the reduced Cu-PSNT had an average size larger than that in the reduced Cu/SiO_2_-AE (Supplementary Fig. 13), what particularly interesting is that, the reduced Cu-PSNT exhibited both much better activity and selectivity than the reduced Cu/SiO_2_-AE (Supplementary Fig. 14). At 200 °C, the reduced Cu-PSNT showed 99.8% conversion of DMO as well as 97.9% selectivity to EG with a LHSV as high as 4.2 h^−1^. In contrast, under the same reaction conditions, the reduced Cu/SiO_2_-AE catalyst gave only 76.2% conversion of DMO and 68.9% selectivity to EG. It should be noted that, even after long time (24 h) catalysis, Cu nanoparticles in the reduced Cu/SiO_2_-AE catalyst did not sinter much and were still smaller than those in the reduced Cu-PSNT catalyst (Supplementary Fig. 13).

The above catalysis comparison between Cu-PSNT and Cu/SiO_2_-AE clearly indicated that the particle size of Cu was not the predominant factor to determine the catalytic performance. The enhanced performance should be attributed to the presence of more abundant Cu–O–SiO_*x*_ interfaces in the reduced Cu-PSNT than Cu/SiO_2_-AE. In principle, the presence of abundant Cu–O–SiO_*x*_ interfaces should result in the presence of more Cu^δ+^ species in the reduced Cu-PSNT. While XPS measurements confirmed the reduction of Cu^2+^ in both reduced Cu-PSNT and Cu/SiO_2_-AE composite (Supplementary Fig. 15), the Cu LMM XAES studies demonstrated that the Cu^+^/Cu^0^ ratios were much different in the two catalysts. Two overlapping peaks at 914.1 eV and 917.8 eV were ascribed to Cu^+^ and Cu^0^, respectively.^28,46,47^ The ratio of Cu^+^/Cu^0^ was 0.65 (Table S1) for the reduced Cu-PSNT, 1.2 times higher than that of the reduced Cu/SiO_2_-AE catalyst (0.55). The high percentage of Cu^+^ confirmed the presence of more Cu–O–SiO_*x*_ interfaces in the reduced Cu-PSNT catalyst, consistent with our proposal that the Cu–O–SiO_*x*_ interface was the determining factor for the catalysis.

### Maximizing both Cu–O–SiO_*x*_ interfaces and Cu utilization

It should be noted that the simple reduction did not maximize the use of Cu due to the formation of some large Cu nanoparticles with size larger than 10 nm (Fig. 4c). There was still possibility to further improve the catalytic performance of Cu-PSNT if one could reduce the particle size of Cu nanoparticles during the H_2_ treatment. To achieve this goal, our strategy was to encapsulate Cu-PSNT with a thin layer of m-SiO_2_. The mesoporous layer SiO_2_ was used to prevent the sintering of Cu nanoparticles during the H_2_ treatment and also to create more Cu–O–SiO_*x*_ interfaces. The coating of the m-SiO_2_ shell was carried out by hydrolysis of TEOS in the presence of CTAB (Fig. 4a, See Methods section)^48^. In the as-obtained core-shell material (denoted as Cu-PSNT@m-SiO_2_), the successful growth of a wormhole-like m-SiO_2_ shell on Cu-PSNT was revealed by TEM analysis (Fig. 4d), and also confirmed by N_2_ adsorption/desorption measurement (Supplementary Fig. 16). Compared with Cu-PSNT, the BET surface area of Cu-PSNT@m-SiO_2_ was increased to 605.5 m^2^ g^−1^.

As expected, the SiO_2_ coating on Cu-PSNT significantly prevented Cu nanoparticles from sintering during the H_2_ treatment as revealed by TEM and XRD studies (Fig. 4e, Supplementary Fig. 17a). After 4-h H_2_ treatment at 300 °C, the XRD and XPS results confirmed the reduction of Cu(II) in Cu-PSNT@m-SiO_2_ into fine fcc Cu nanoparticles (Supplementary Figs. 15 and 17a). The yielded Cu nanoparticles were even too small to be clearly detected by TEM and STEM (Fig. 4e and Supplementary Fig. 17b) due to the limited electronic contrast between Cu and SiO_2_. No formation of large Cu nanoparticles with size larger than 2 nm was observed in the reduced Cu-PSNT@m-SiO_2_ catalyst, dramatically different from the reduced Cu-PSNT. As determined by ICP-AES, the Cu content in Cu-PSNT@m-SiO_2_ was as high as 20.5 wt% (Supplementary Table 2). More importantly, the ratio of Cu^+^/Cu^0^ demonstrated by Cu LMM XAES spectra is 0.99 (Supplementary Fig. 15 and Supplementary Table 1), 1.5 and 1.8 times higher than those of Cu-PSNT and Cu/SiO_2_–AE catalysts. These results suggested that Cu in reduced Cu-PSNT@m-SiO_2_ were present mainly in the form of ultrafine nanoparticles confined in the SiO_2_ matrix. The confinement of fine Cu nanoparticles in porous SiO_2_ was expected to create abundant Cu–O–SiO_*x*_ interfaces to boost the hydrogenation. In situ FT-IR measurements over the reduced Cu-PSNT@m-SiO_2_ catalyst under D_2_ atmosphere revealed the formation of SiO-D (Supplementary Fig. 18), further confirming the heterolytic activation pathway of D_2_ over the Cu–O–SiO_*x*_ interfaces.

As compared to the reduced Cu-PSNT catalyst with the same amount of Cu, the catalytic performance of the reduced Cu-PSNT@m-SiO_2_ catalyst was greatly enhanced in both activity and selectivity for DMO hydrogenation to EG. As shown in Fig. 4f, a nearly 100 % conversion of DMO and a high selectivity of 96.0% to EG were achieved over the reduced Cu-PSNT@m-SiO_2_ catalyst even at a LHSV as high as 7.8 h^−1^ at 200 °C. Under the same catalytic conditions (high LHSV), the reduced Cu-PSNT only gave 33.7% conversion of DMO and 22.6% selectivity to EG. The TOF (Supplementary Table 2) of the reduced Cu-PSNT@m-SiO_2_ catalyst (40.62 h^−1^) was much higher than that of the reduced Cu-PSNT (23.08 h^−1^) or Cu/SiO_2_–AE (10.21 h^−1^) catalyst. More importantly, after catalysis studies at different LHSVs, no formation of large Cu nanoparticles caused by sintering was observed over the reduced Cu-PSNT@m-SiO_2_ catalyst (Supplementary Fig. 19). The reduced Cu-PSNT@m-SiO_2_ also displayed excellent stability in the time-on-stream experiment (Fig. 4g). No decay in the activity and stability of the reduced Cu-PSNT@m-SiO_2_ catalyst was observed even after the 200 h time-on-stream (LHSV = 2.0 h^−1^) experiment. Additionally, the reduced Cu-PSNT@m-SiO_2_ also displayed excellent stability in ultra-high temperature and ultra-high LHSV (Supplementary Fig. 20). At 280 °C, the reduced Cu-PSNT@m-SiO_2_ exhibited ~95% conversion of DMO as well as ~90% selectivity of EG with a LHSV as high as 300 h^−1^ during the long time (16 h) catalysis. To the best of our knowledge, the catalytic activity of the reduced Cu-PSNT@m-SiO_2_ showed the best performance among reported copper-based catalysts for ester hydrogenation (Supplementary Table 3).

Although SiO_2_ has been long considered as an inert support to create active metal-support interface for promoting catalysis, we demonstrate in this work that Cu–O–SiO_*x*_ is a very active interface in selective hydrogenation of DMO to EG. The activity of silica-coated Cu catalysts with Cu–O–SiO_*x*_ interfaces in selective hydrogenation of DMO is approximately 80 times higher than that on pristine Cu at the temperature between 200 and 240 °C. With the combination of DFT calculations and isotope-labelling experiments, the catalytic mechanism on Cu–O–SiO_*x*_ interface has been well clarified. The existence of Cu-H^δ−^ and SiO-H^δ+^ at Cu–O–SiO_*x*_ interfaces would facilitate the hydrogenation of ester by stablizing the hydrogenation transition states. Based on this mechanism, we develped a confined growth strategy to maximize Cu–O–SiO_*x*_ interfaces and Cu utilization. By coating copper phyllosilicate nanotubes with mesoporous silica followed by hydrogen reduction, a practical Cu nanocatalyst was produced and possessed abundant Cu–O–SiO_*x*_ interfaces and thus exhibited the best performance in the hydrogenation of DMO to EG among all reported Cu catalysts. We envision that the discovery of the active Cu–O–SiO_*x*_ interface for promoting catalysis in this work will lead us to revisit the support effects of SiO_2_ and create pratical SiO_2_-supported metal nanocatalysts with enhanced catalytic activity, selectivity, and durability.

## Methods

### Materials

Colloidal silica (Ludox-HS 40, SiO_2_ 40 wt % aqueous solution), methanol and tetraethyl orthosilicate were purchased from Alfa Aesar Chemical Reagent Co. Ltd. (Tianjin, China). Cu(NO_3_)_2_·3H_2_O, DMO, CuCl_2_·2H_2_O, ammonium chloride, ethanol, N-hexadecyltrimethylammonium bromide and ammonia aqueous solution (25%~28%) were purchased from Sinopharm Chemical Reagent Co. Ltd. (Shanghai, China). Cu powder was purchased from Tianjin Guangfu Fine Chemical Co., Ltd. Deuterium gas (99.999%) was purchased from Chengdu Keyuan Gas Co. Ltd. All reagents were used as received without further purification. Water used in the studies was ultrapure water (Millipore, ≥18 MΩ cm).

### Synthesis of cuprous oxide nanoparticles (Cu_2_O NPs)

Cu_2_O NPs were prepared by using the high-temperature ploy-mediated methods reported by Zeng^49^. In a typical synthesis of Cu_2_O NPs, 0.5 mmol of Cu(NO_3_)_2_·3H_2_O and 1.0 g of PVP were dissolved in 10 mL of DEG. The mixed solution was heated from room temperature to 190 °C in 0.5 h under argon atmosphere. The products were collected by centrifugation and washed with ethanol for several times. Finally, the products were dispersed in ethanol for further use.

### Synthesis of Cu_2_O NPs coated with mesoporous silica

Cu_2_O@m-SiO_2_ were prepared by using the modified method reported by Fang^50^. In a typical synthesis of Cu_2_O@m-SiO_2_, 0.2 g of Cu_2_O NPs and 1 g of CTAB were added into 200 mL water in flask under argon atmosphere and then transferred to a 45 °C water bath. Then, 5 mL of ethanol and 0.4 mL of TEOS were added into above mixture and stirred for another 30 min. The products were collected by centrifugation and washed with ethanol several times. The method of extraction was used to remove CTAB from the products. Briefly, the products were dispersed in 100 mL of acetone and refluxed at 80 °C for 8 h. The extraction was repeated three times to fully remove CTAB. Finally, the products were collected by centrifugation and washed with ethanol several times.

### Synthesis of copper phyllosilicate nanotubes

Copper phyllosilicate nanotubes (Cu-PSNT) were prepared by a hydrothermal method. Typically, 6.5 mmol of copper (II) salt (CuCl_2_·2H_2_O or Cu(NO_3_)_2_·3H_2_O) and 26 mmol of NH_4_Cl were dissolved in 60 mL water, into which 5 mL of NH_3_·H_2_O was added to form a blue solution. Then, 1 g of silica colloidal (SiO_2_ 40 wt %) was added into above solution. Subsequently, the mixture was transferred into 100 mL capacity Teflon-lined stainless steel autoclave and then the autoclave was put in an oven at 200 °C for 48 h. The blue products were collected by centrifugation and washed with water for several times. Finally, the blue products were dried in a vacuum oven at 60 °C for 12 h.

### Synthesis of Cu-PSNT coated with mesoporous silica

Cu-PSNT@m-SiO_2_ was prepared using the modified method reported by Fang^50^. For a typical synthesis of Cu-PSNT@m-SiO_2_, 0.6 g of copper phyllosilicate nanotubes was dispersed into 200 mL water containing 1 g of CTAB in the flask. The mixture was transferred to a 45 °C water bath and then ethanol (5 mL) and TEOS (2 mL) were added. After 30 min, the products were collected by centrifugation and washed with ethanol several times. Subsequently, the products were dried in a vacuum oven at 60 °C for 12 h. Calcination was used to remove CTAB and the products were heated to 500 °C (2 °C/min) for 2 h in air.

### Synthesis of the Cu/SiO_2_-AE catalyst

Cu/SiO_2_-AE was prepared by ammonia evaporation method^38^. The ammonia evaporation method was described as follows: 3.05 g of Cu(NO_3_)_2_·3H_2_O was dissolved in a mixture of ultrapure water (75 mL) and ammonia aqueous (5 mL). Then, 20 g of silica colloidal (SiO_2_ 40 wt %) was added into above copper ammonia complex solution. Subsequently, the mixed solution was heated in an 85 °C water bath to evaporate ammonia. As the process continues, the pH value of the mixture decreased slowly. When the pH value decreased below 7.0, the products were collected by centrifugation and washed with water for several times. Finally, the products were dried in an oven at 60 °C for 12 h and then were heated to 500 °C (2 °C per min) for 2 h in air.

### Characterizations

Transmission electron microscopy (TEM) images were taken on a TECNAI F-30 high-resolution transmission electron microscope operating at 300 kV. The conventional and in situ X-ray powder diffraction (XRD) were performed with PANalytical X’pert PRO diffractometer using Cu K_α_ radiation (*λ* = 0.15418 nm), operating at 40 kV and 30 mA. For the in situ XRD measurement, the samples were put in an in situ chamber and 5%H_2_-95%N_2_ mixture gas was introduced to the system at a flow rate of 50 mL per min. Then, the sample was heated to 573 K (2 °C per min) for 4 h. When the temperature of sample cooled to room temperature, the XRD patterns were collected. N_2_ adsorption–desorption measurements were carried on a Micrometrics ASAP 2020 system. Pore size distributions were calculated from desorption branch by the Barrett-Joyner-Halenda (BJH) method. The total pore volume depended on the desorption N_2_. X-ray photoelectron spectroscopy (XPS) and X-ray induced Auger electron spectroscopy (XAES) were obtained using a PHI Quantum 2000 Scanning ESCA Microprobe instrument (physical Electronics) equipped with an Al Kα X-ray source (*hν* = 1486.6 eV) and binding energies referenced to C 1 s (284.8 eV). For quasi-in situ XPS measurement, the samples were treated with 5%H_2_-95%N_2_ (573K-4 h) in an in situ chamber, and then evacuated to obtain a high vacuum environment. Finally, the reduced samples were transferred from in situ chamber to testing chamber under vacuum conditions. The precise copper content of sample was determined by the inductively coupled plasma atomic emission spectroscopy (ICP-AES, Baird PS-4). The copper dispersions of the samples were measured by N_2_O titration on a Micromeritics Autochem II 2920 apparatus with a TCD. Typical steps as follows: (1) Samples were reduced in a 5%H_2_-95%N_2_ atmosphere (50 mL per min) at 573 K for 4 h (hydrogen consumption was denoted as *A*_1_) and then cooled down to 333 K in argon atmosphere. (2) The surface copper atoms were oxidized to Cu_2_O by N_2_O (30 mL/min) for 0.5 h and then the argon was introduced to the system for 0.5 h to remove the N_2_O. (3) The reduction of surface Cu_2_O to copper was carried out by a 5%H_2_-95%N_2_ mixture gas (50 mL per min) at 773 K for 2 h (hydrogen consumption was denoted as *A*_2_). The dispersion (*D*) of copper was calculated by *D* = (2*A*_2_/*A*_1_)*100%.

### Catalytic performance tests

The catalytic performance for DMO hydrogenation was evaluated by using a fixed-bed microreactor. Typically, 200 mg of the catalyst was placed in the middle of the quartz tube and packed with quartz powders in the top side. The quartz tube was then loaded into the stainless steel tubular reactor. The catalyst was reduced under a 5%H_2_-95%N_2_ flow (50 mL per min) at 573 K (2 °C per min) for 4 h. The catalyst was cooled to desired reaction temperature (473 K). Subsequently, 10 wt % DMO in methanol and H_2_ were fed into the reactor at a H_2_/DMO molar ratio of 80 under a system pressure of 3.0 MPa. The liquid hourly space velocity of DMO was varied by changing the amount of feedstock. The outlet stream was sampled by an automatic Valco 6-ports valve system and analyzed by an online gas chromatograph (GC-9790, FuLi) with a flame ionization detector and a KB-Wax capillary column (30 m × 0.45 mm × 0.85 μm) at intervals of 0.5 h.

### Computational details

DFT calculations are carried out by using the Vienna ab initio simulation package (VASP)^51,52^. Exchange and correlation were treated within the Perdew-Burke-Ernzerhof (PBE) generalized gradient approximation (GGA)^43^.The valence electrons are described by plane wave basis sets with a cut off energy of 400 eV, and the core electrons are replaced by the projector augmented wave pseudopotentials^53,54^. For a clean Cu(111) surface, a (5 × 3) supercell with five layer slabs was used, which for the SiO_2_/Cu(111), the Cu(111) substrate was extended into the (8 × 4) structure. During structural optimization, the bottom two layer slabs were fixed at a bulk truncated position, while the surface layers and the adsorbates were fully relaxed. For all of the calculations, the vacuum regions between the slabs were more than 10 Å, and Monkhurst-Pack k-point sampling with approximately 0.05 × 2π Å^−1^ spacing in a reciprocal lattice was utilized. The minimum energy reaction pathways were calculated using the nudged elastic band method. The final transition state structures were refined using a quasi-Newton algorithm until the Hellman-Feynman forces on each ion were lower than 0.03 eV Å^−1^. The adsorption energies (Δ*E*_ads_) were calculated using Eq. 1, in which *E*_ad/surf_, *E*_ad_, and *E*_surf_ were the total energies of the optimized adsorbate/surface system, the adsorbate in the gas phase, and the surface respectively. For the DMO hydrogenation, we assumed that there already existed four adsorbed H atoms on the Cu(111) surface and SiO_2_/Cu(111) interface.

1 $$\Delta {E}_{{\mathrm{ads}}} = {E}_{{\mathrm{ad/surf}}}-{E}_{{\mathrm{ad}}}-{E}_{{\mathrm{surf}}}$$

### Data availability

The data that support the findings of this study are available from the corresponding author upon reasonable request.

## Electronic supplementary material

Supplementary Information
