# Supplementary material for: Interfacing with silica boosts the catalysis of copper
Source: Nat Commun. 2018 Aug 22;9:3367. doi: 10.1038/s41467-018-05757-6 (PMC6105597; doi:10.1038/s41467-018-05757-6)
Supplement: Supplementary file 1 — Supplementary Information [file 41467_2018_5757_MOESM1_ESM.pdf]

Supplementary Materials for

Interfacing with Silica Boosts the Catalysis of Copper

Chaofa Xu,[1,+] Guangxu Chen,[1,+] Yun Zhao,[1] Pengxin Liu,[1] Xinping Duan,[1] Lin Gu,[2] Gang Fu,[1,*] Youzhu Yuan,[1,*] and Nanfeng Zheng [1,*]

1. State Key Laboratory for Physical Chemistry of Solid Surfaces, Collaborative Innovation Center of Chemistry for Energy Materials, and National Engineering Laboratory for Green Chemical Productions of Alcohols–Ethers–Esters, College of Chemistry and Chemical Engineering, Xiamen University, Xiamen 361005, China

2. Institute of Physics, Chinese Academy of Sciences, Beijing 100190, China

[+] These authors contributed equally to this work.

* Corresponding authors. E-mail: nfzheng@xmu.edu.cn (N. F. Zheng); gfu@xmu.edu.cn (G. Fu); yzyuan@xmu.edu.cn (Y. Z. Yuan)

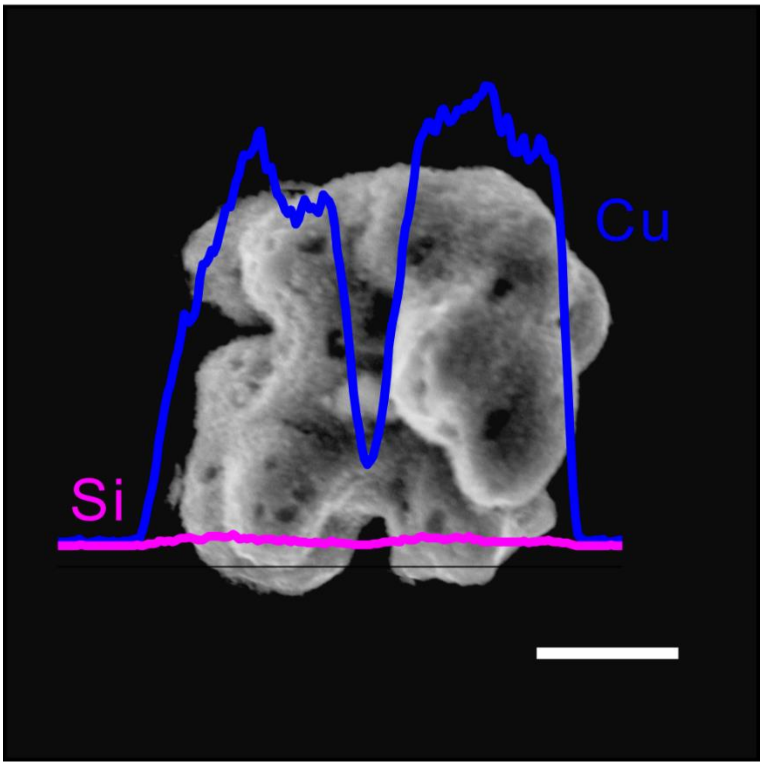

Supplementary Figure 1. SEM image and EDS line profile of Cu MP@m-SiO_2. Scale bar is 2um for the image.

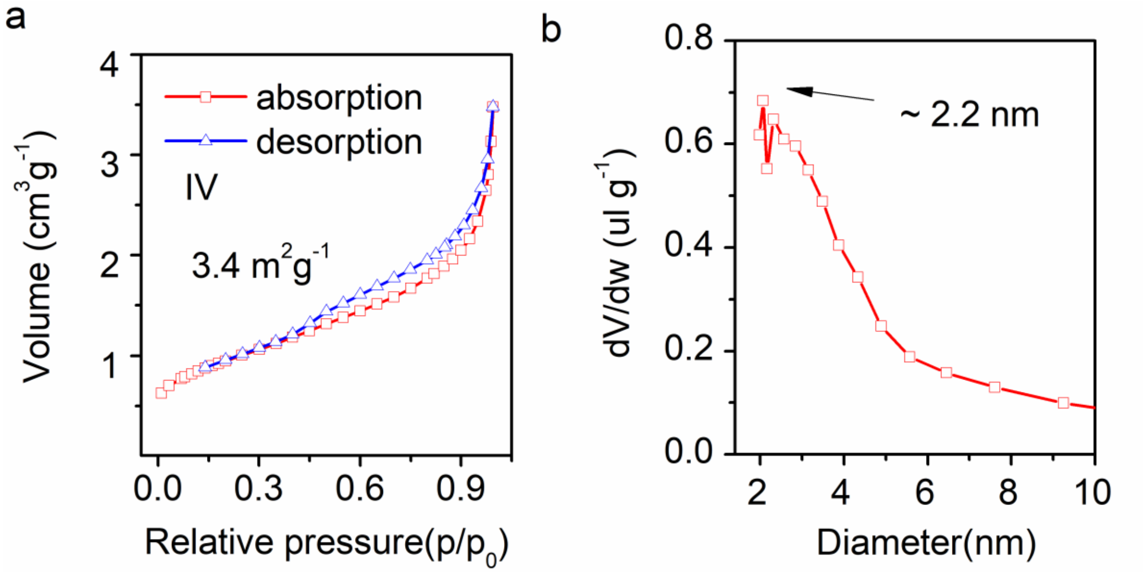

Supplementary Figure 2. Porosity measurements of Cu MP@m-SiO_2. **a**, N_2 adsorption/desorption isotherm; **b,** the pore-size distribution curve obtained from the desorption data of Cu MP@m-SiO_2.

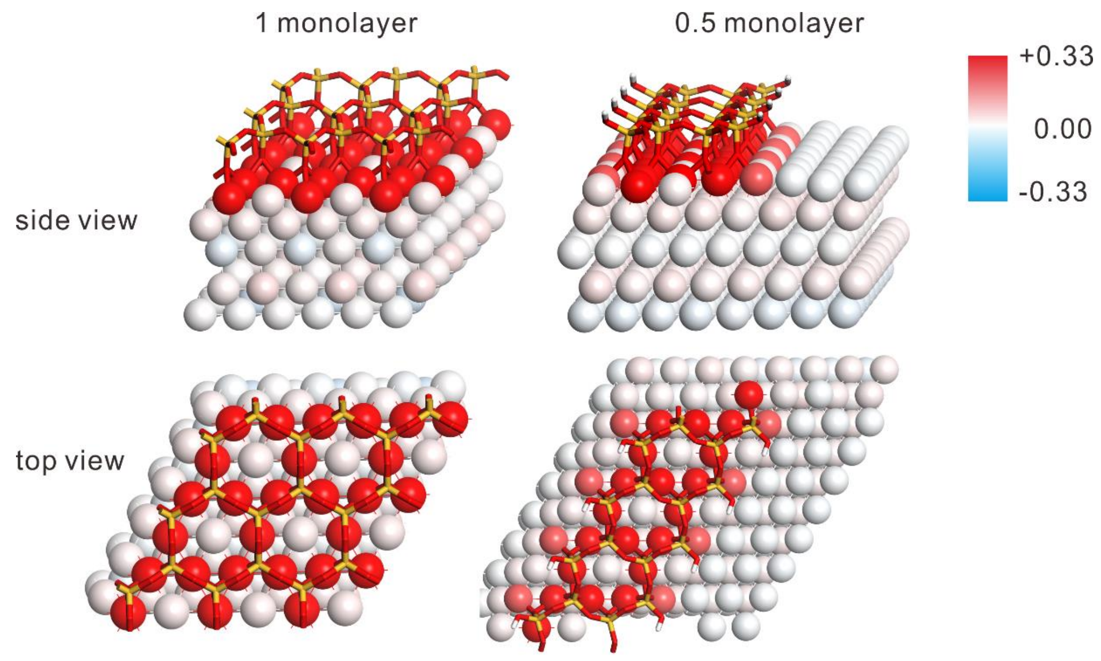

Supplementary Figure 3. The structural models of periodic Cu (111) with SiO_2 coating and Bader charge analysis of surface Cu atoms.

ΔE_{bind}=[E(Cu（111）/SiO_2)-2/3E(SiO_2_quartz)-1/2E(O_2)-E(Cu（111））]/2= -0.66 eV Si^{-1}

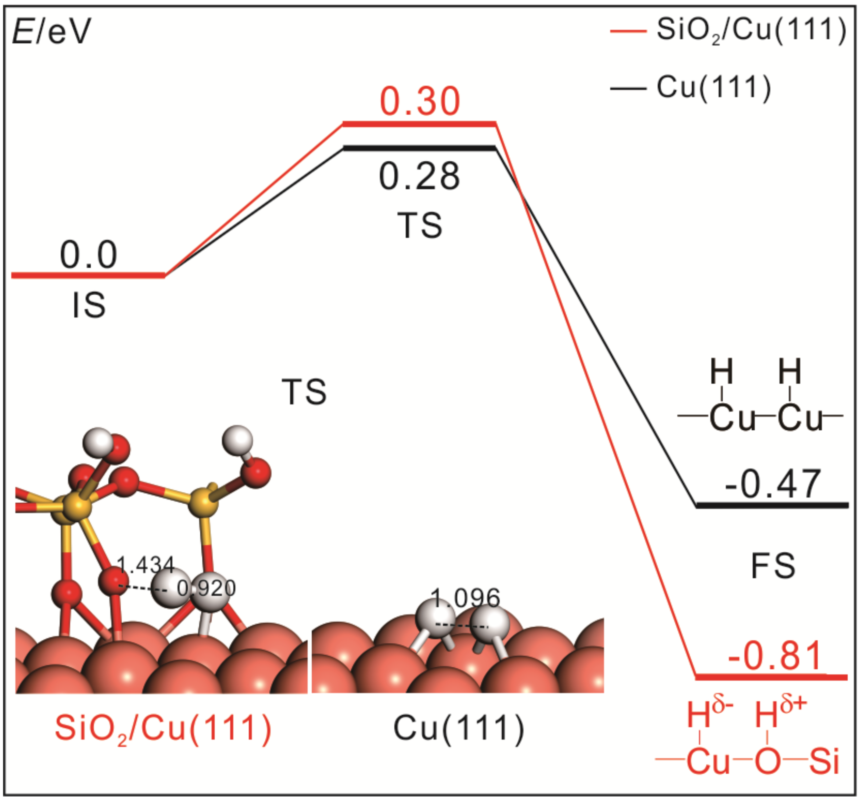

Supplementary Figure 4. The dissociation of H_2 on the surfaces of Cu(111) and SiO_2/Cu(111).

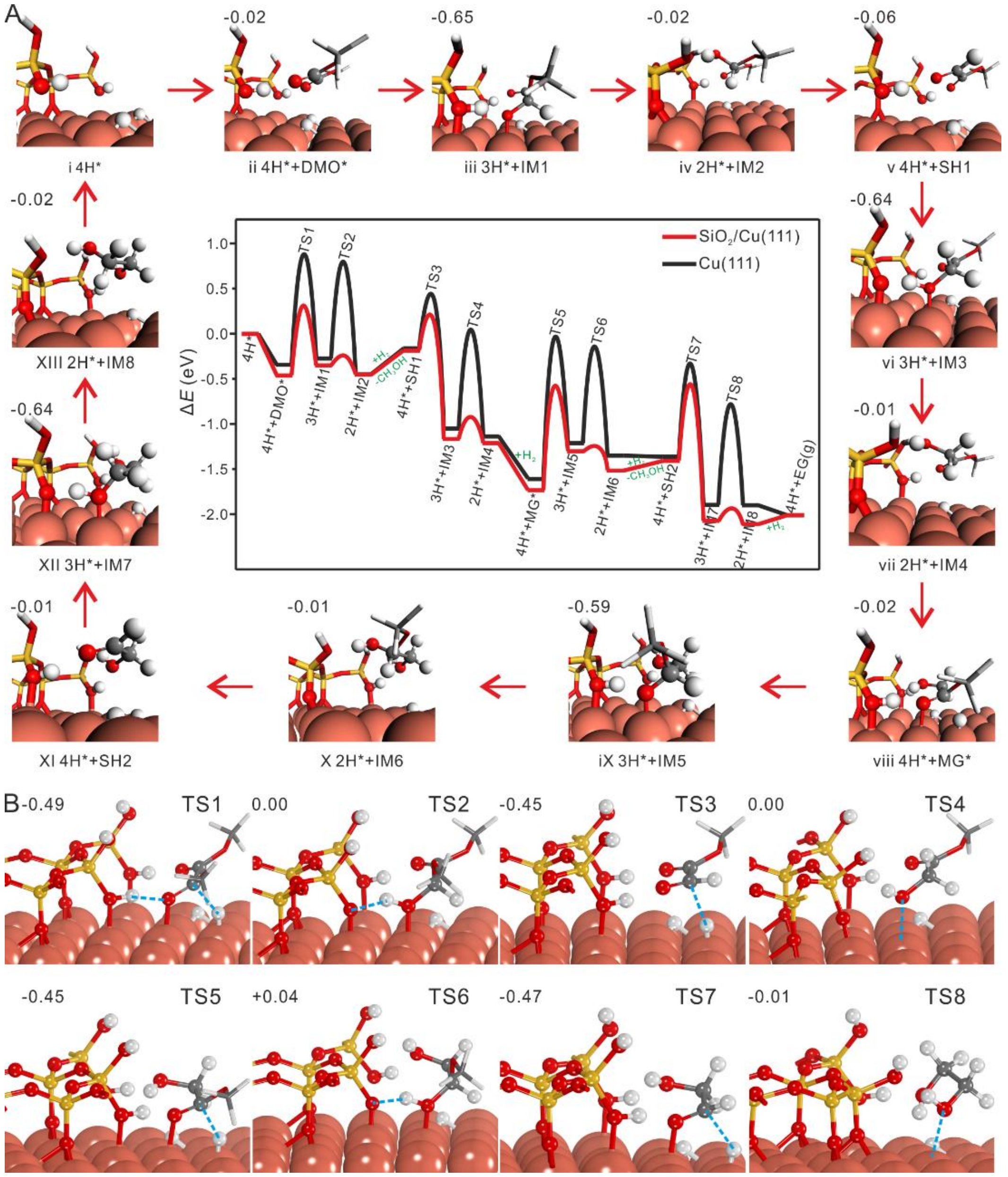

Supplementary Figure 5. Mechanism on how the Cu-O-SiO_x interface promotes DMO hydrogenation. **a**, The DMO hydrogenation pathway on Cu(111) and SiO_2/Cu(111); **b**, The optimized structures of transition states (TS) and important intermediates (IMs) of SiO_2/Cu(111). The number listed in the upper right corner was the Bader charge of reactant or hydrogenated intermediate.

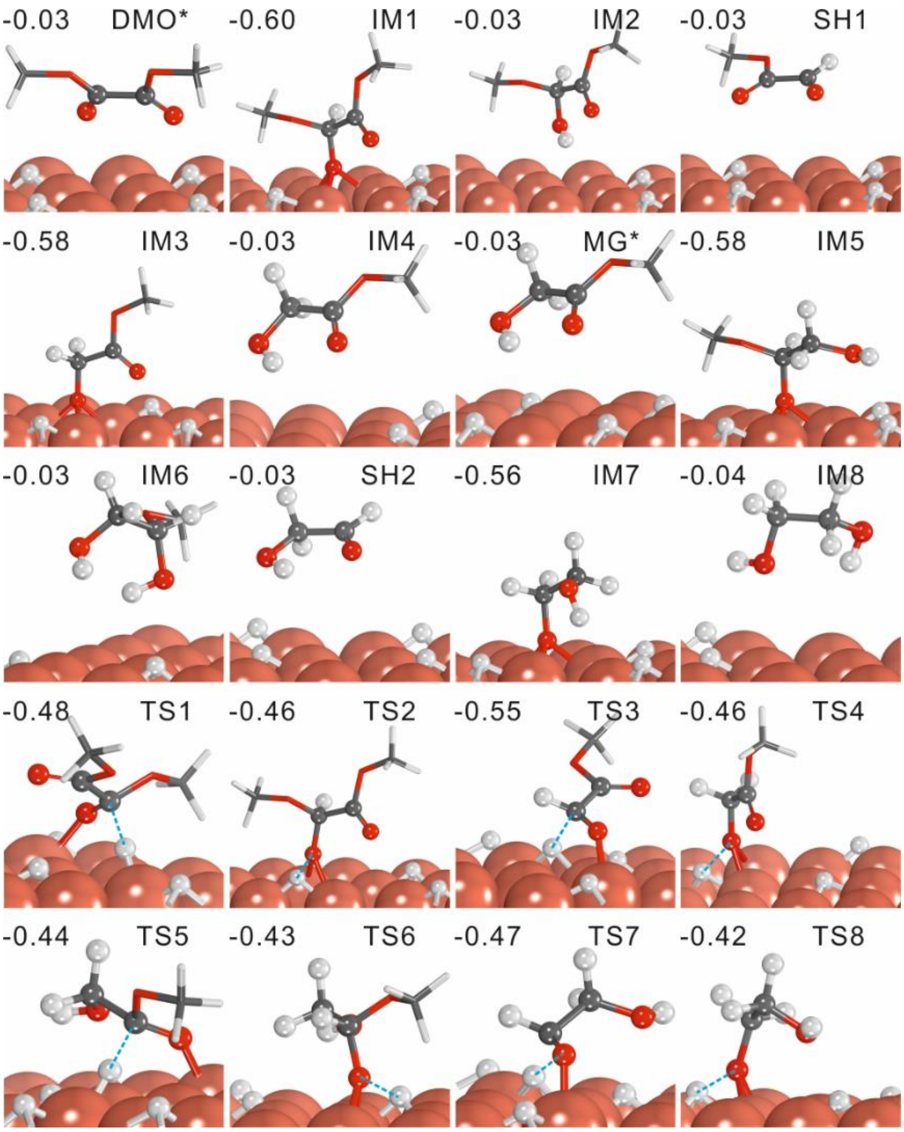

Supplementary Figure 6. The optimized structures of transition states (TS) and important intermediates (IMs) of Cu(111). The number listed in the upper right corner was the Bader charge of reactant or hydrogenated intermediate.

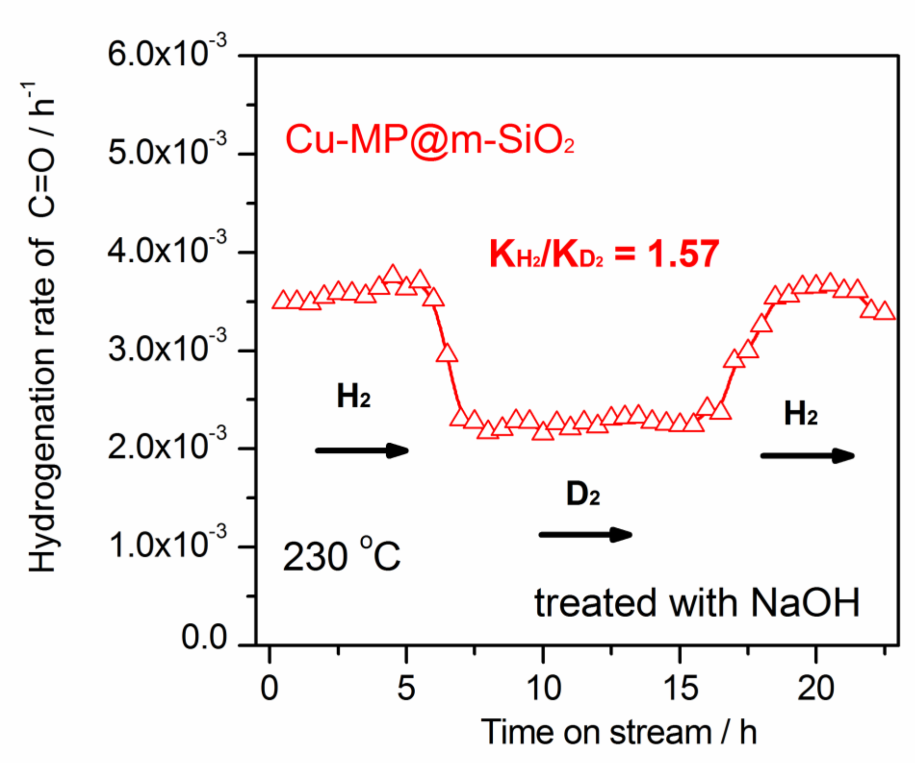

Supplementary Figure 7. The kinetic isotope effect of Cu-MPs@m-SiO_2 catalyst after treating with NaOH (0.1M). Reaction conditions were as follows: H_2 / DMO = 80 mol/mol, P (H_2) = 3.0 MPa, T = 230 ºC.

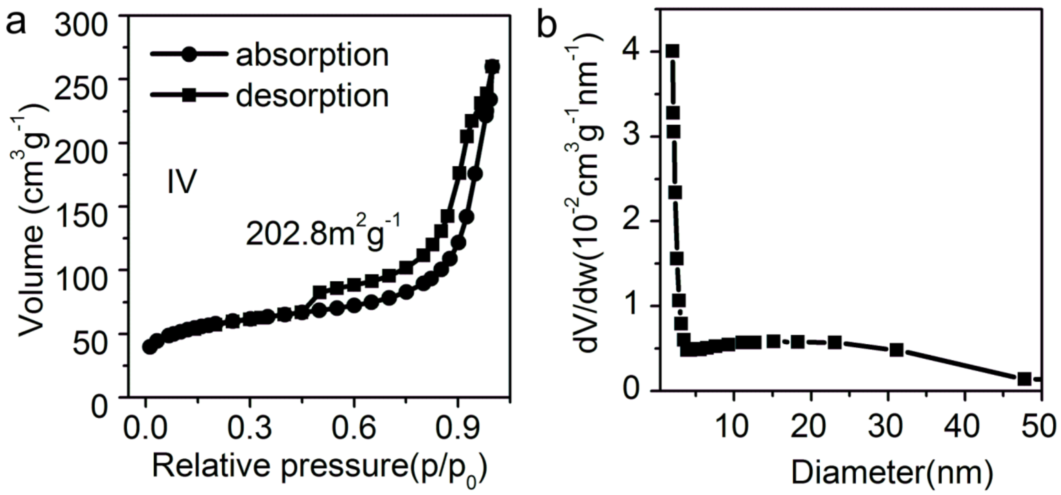

Supplementary Figure 8. Porosity measurements of Cu_2O@m-SiO_2. **a**, N_2 adsorption/desorption isotherm; **b**, the pore-size distribution curve obtained from the desorption data of Cu_2O@m-SiO_2; Pore size distribution was calculated from the desorption branch by the Barrett-Joyner-Halenda (BJH) method.

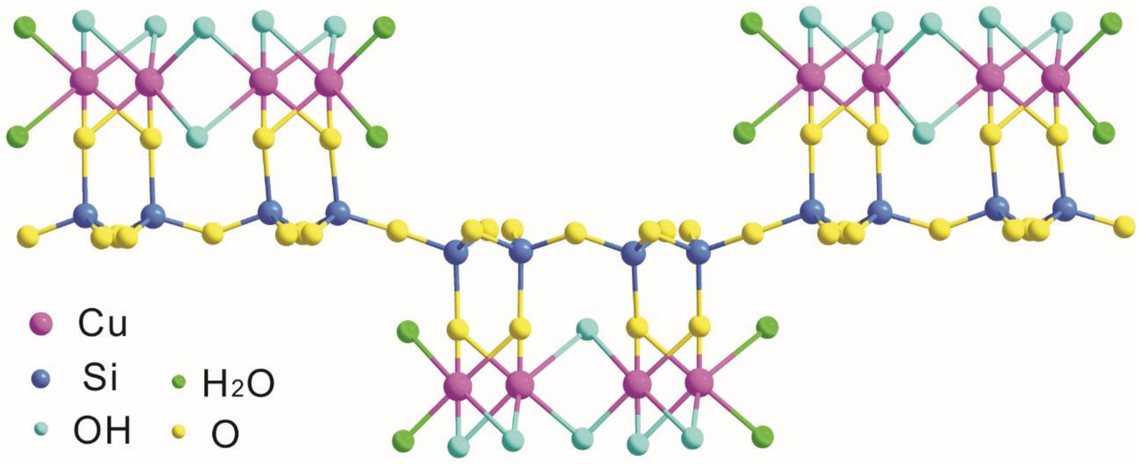

Supplementary Figure 9. Schematic representation of the structure of bulk copper phyllosilicate. Copper phyllosilicate ($Cu_{2-x}Si_2O_5(OH)_3 \cdot xH_2O$), also called chrysocolla, consists of alternate layer of SiO_4 tetrahedra and discontinuous layers of CuO_6 octahedra.

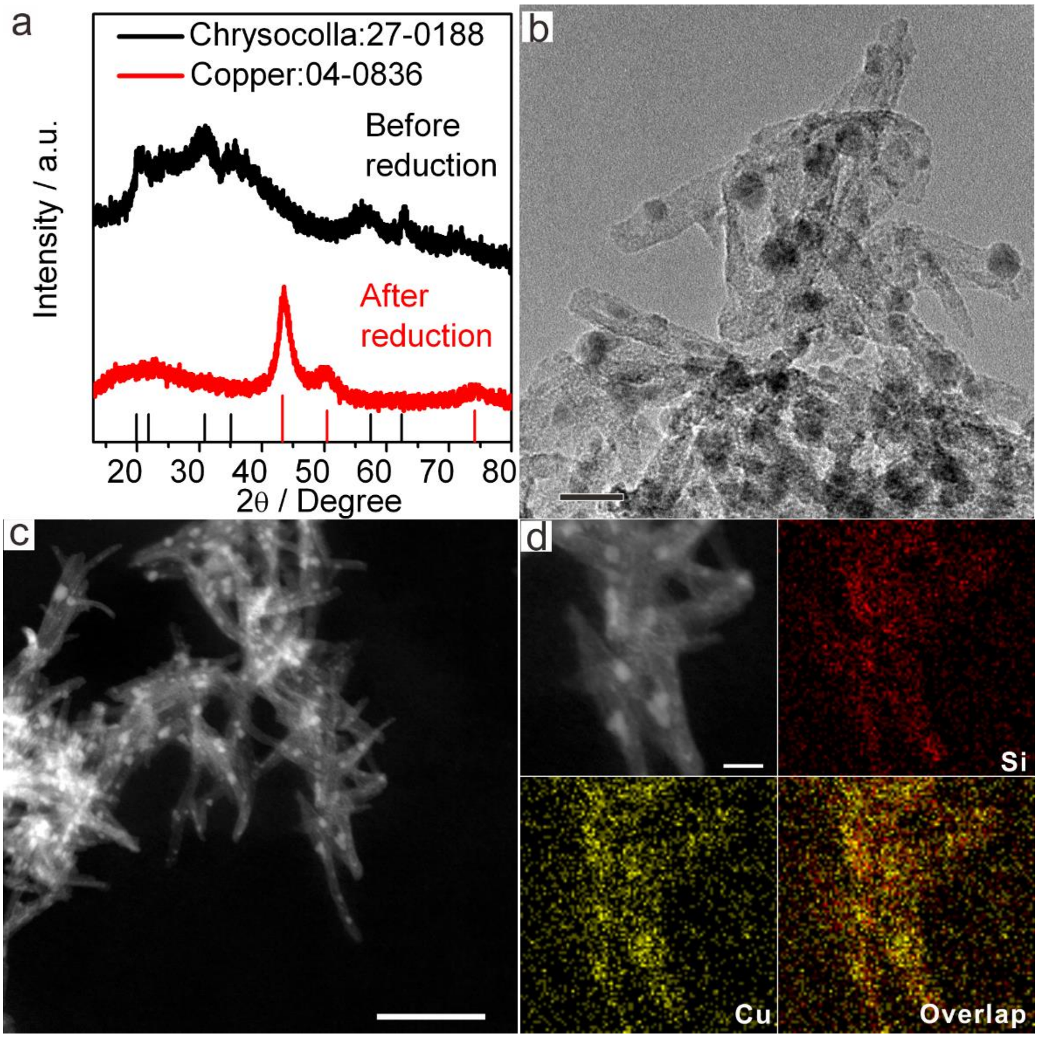

Supplementary Figure 10. Characterizations of Cu-PSNT. **a**, XRD patterns of copper phyllosilicate nanotubes (Cu-PSNT) before and after in situ reduction; **b**, TEM image of reduced Cu-PSNT; **c**, **d**, Representative high-angle annular-dark-field (HAADF) STEM image and EDX mapping images of reduced Cu-PSNT, respectively. Scale bars in b, c and d are 20nm, 100nm and 10nm, respectively.

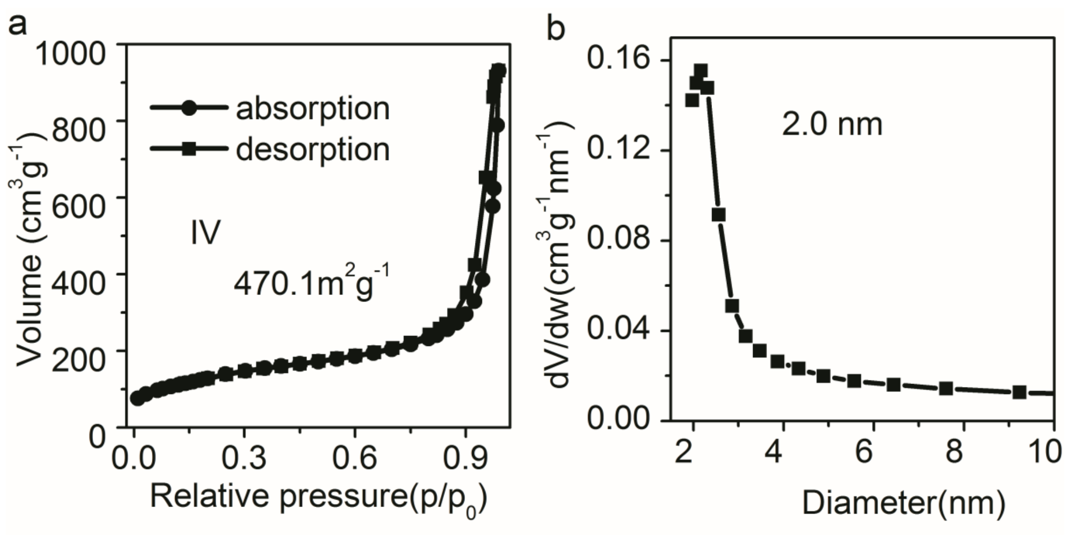

Supplementary Figure 11. Porosity measurements of reduced Cu-PSNT. **a**, N_2 adsorption/desorption isotherm; **b**, the pore-size distribution curve obtained from the desorption data of reduced Cu-PSNT.

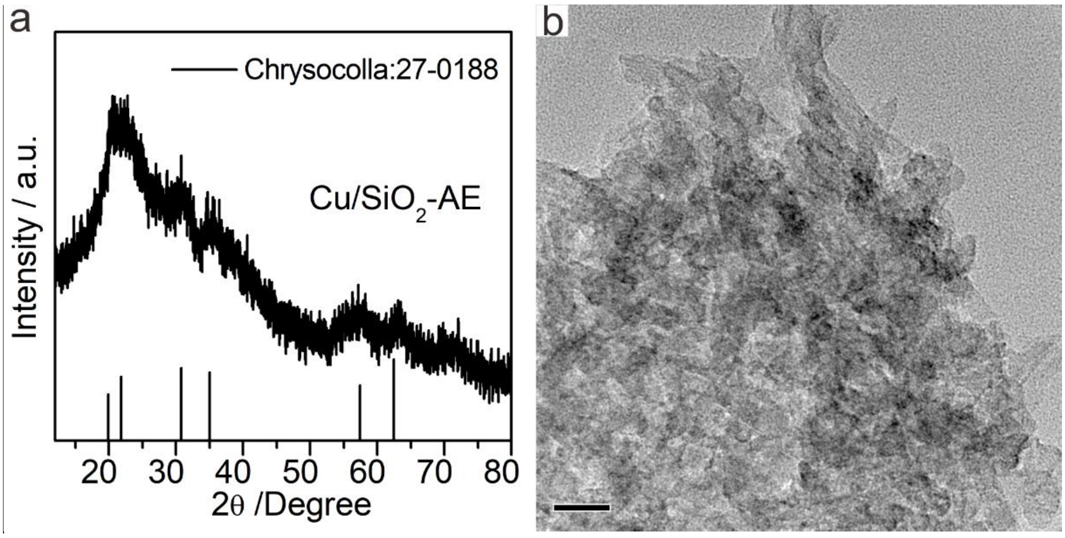

Supplementary Figure 12. Characterizations of Cu/SiO_2-AE before catalysis. **a** , X-ray powder diffraction pattern; **b**, TEM image of as-prepared Cu/SiO_2-AE (AE method). Scale bar in b is 20 nm.

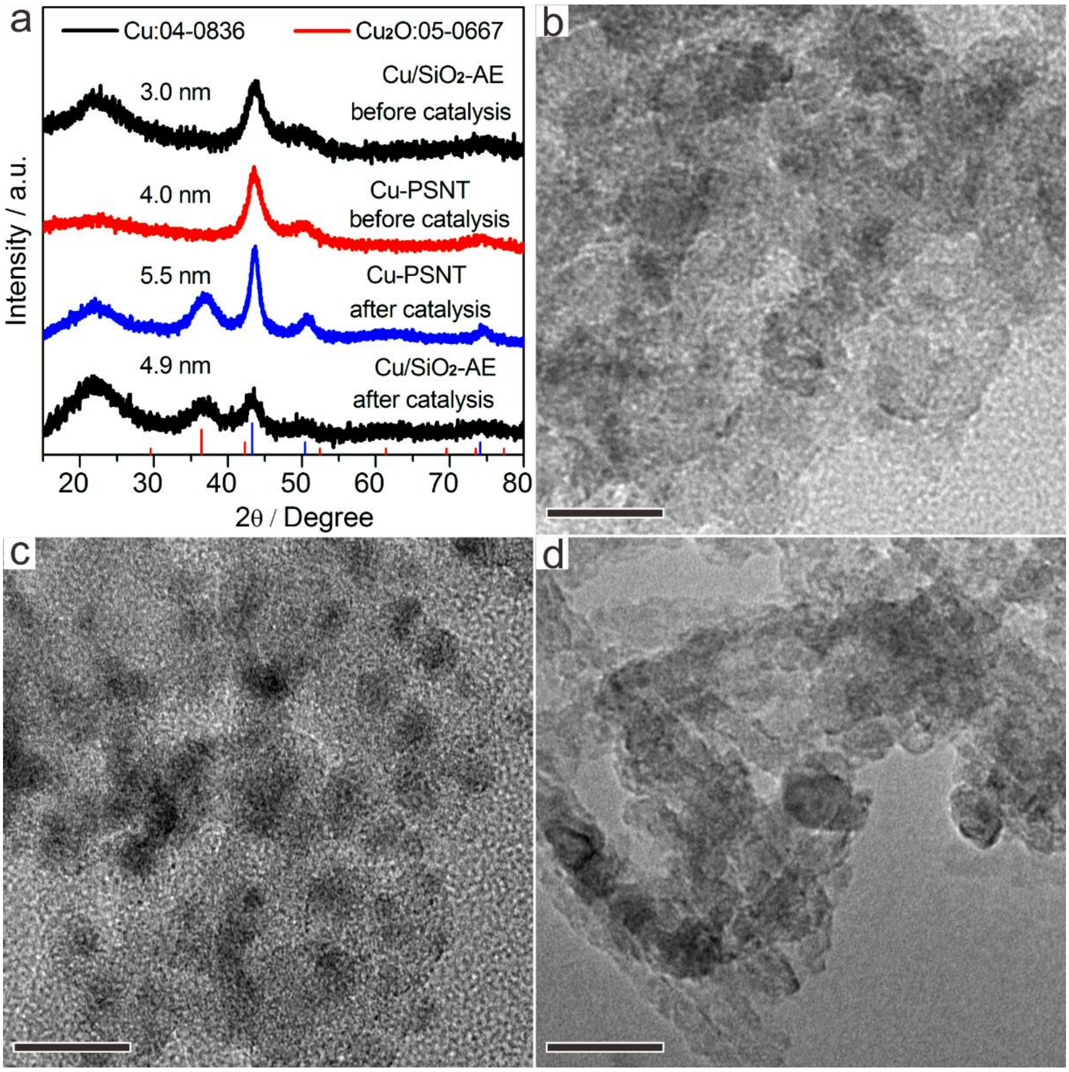

Supplementary Figure 13. Characterizations of Cu/SiO_2-AE catalyst. **a**, X-ray powder diffraction patterns of copper-based catalysts before and after catalysis; **b-d**, TEM images of Cu/SiO_2-AE before catalysis (reduced), Cu/SiO_2-AE after catalysis and Cu-PSNT catalyst after catalysis, respectively. Scale bars are 20 nm for all panels b-d.

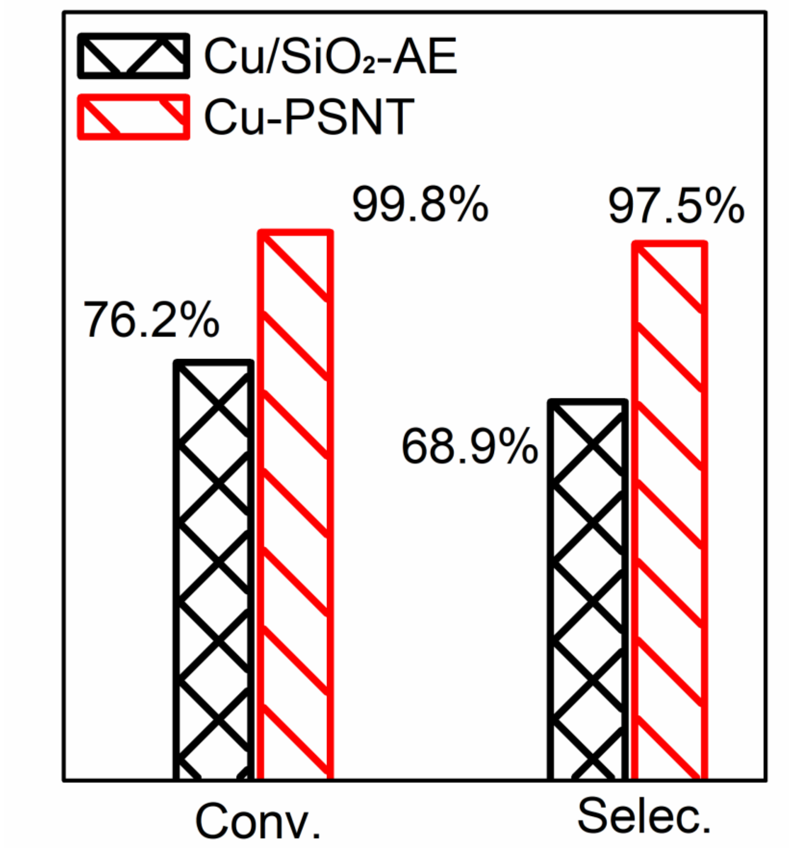

Supplementary Figure 14. Catalytic performance of catalysts. Reaction conditions were as follows: H_2 / DMO = 80 mol/mol, P (H_2) = 3.0 MPa, T = 200 °C, LHSV = 4.2 h^{-1}.

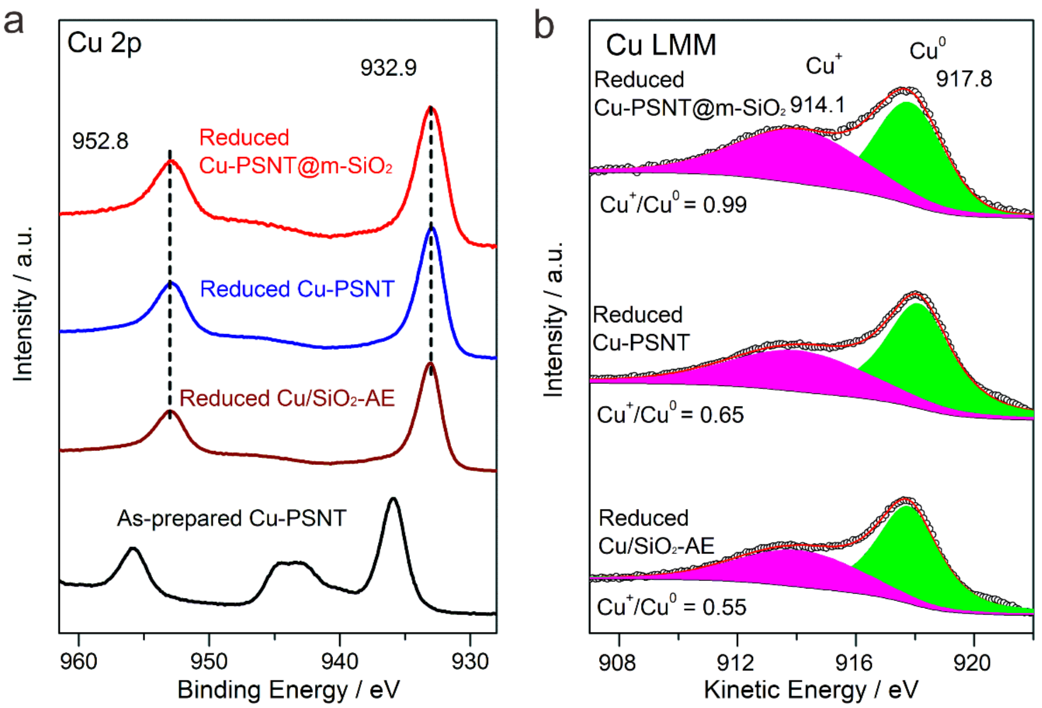

Supplementary Figure 15. The XPS characterizations of catalysts (*quasi-in-situ*). **a**, Cu 2p XPS of the reduced Cu/SiO_2-AE, the reduced Cu-PSNT and the reduced Cu-PSNT@m-SiO_2, respectively; **b**, Cu LMM XAES spectra of Cu-PSNT, the reduced Cu/SiO_2-AE, the reduced Cu-PSNT and the reduced Cu-PSNT@m-SiO_2, respectively.

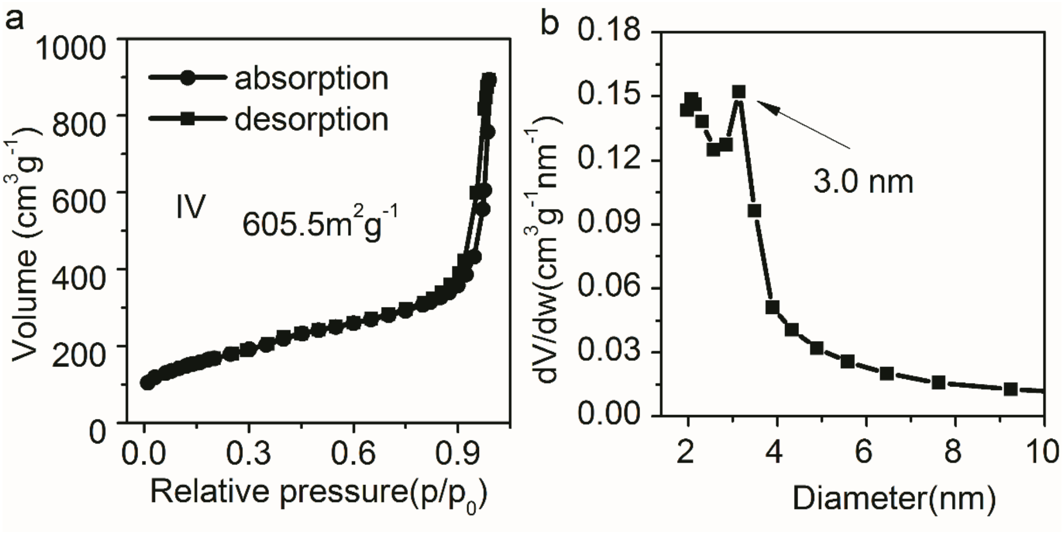

Supplementary Figure 16. Porosity measurements of Cu-PSNT@m-SiO_2. **a**, N_2 adsorption/desorption isotherms; **b**, the pore-size distribution curve obtained from the desorption data of Cu-PSNT@m-SiO_2.

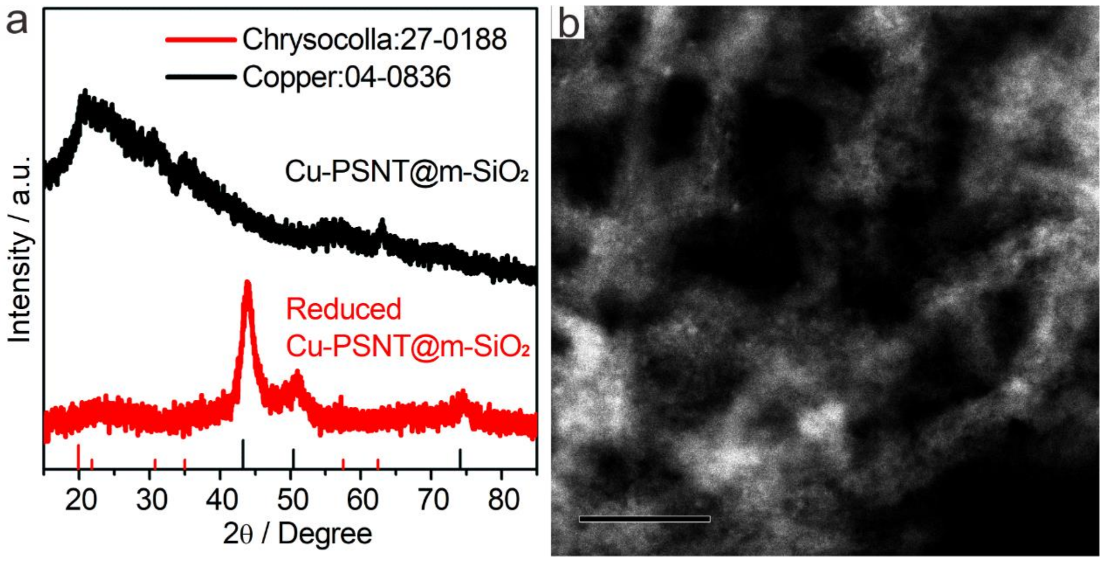

Supplementary Figure 17. Structure characterizations of Cu-PSNT@m-SiO_2. **a**, X-ray powder diffraction patterns of Cu-PSNT@m-SiO_2 before and after in situ reduction; **b**, Representative high-angle annular-dark-field (HAADF) STEM image of the reduced Cu-PSNT@m-SiO_2. Scale bar in b is 50 nm.

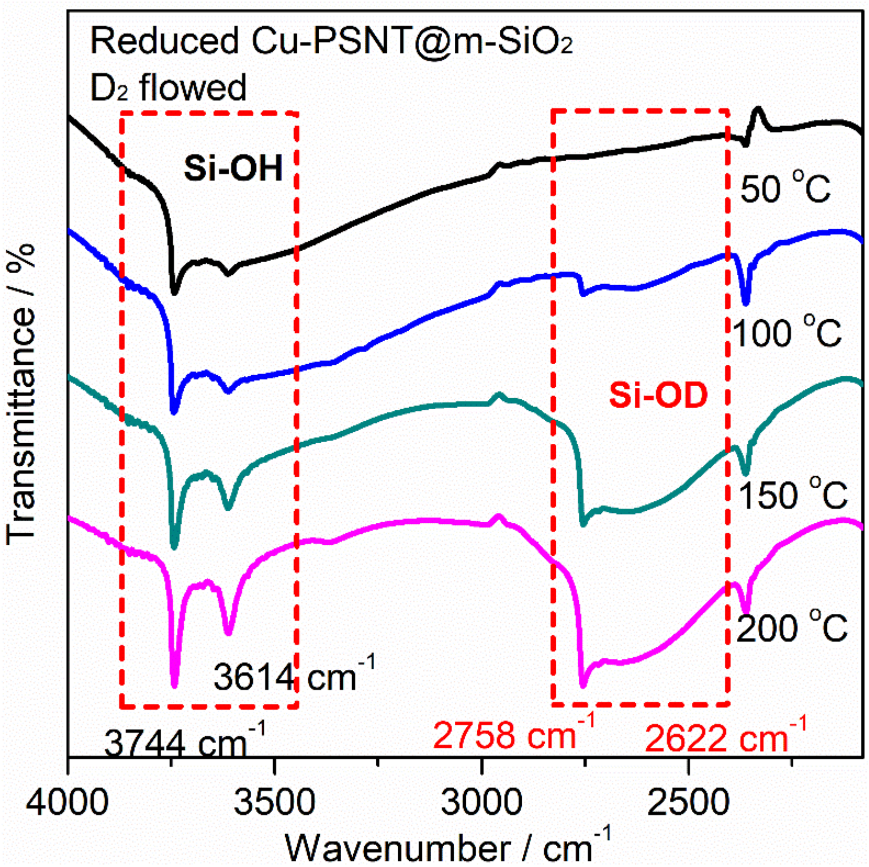

Supplementary Figure 18. In situ FT-IR measurement over the reduced Cu PSNT@m-SiO_2 catalyst and pristine SiO_2 under D_2 atmosphere. The in situ IR spectra were recorded on a Nicollet iS50 FT-IR spectrometer with diffuse reflection mode. In a typical testing, 25 mg of the catalyst was placed in an in-situ cell and reduced under a 5% H_2 + 95% N_2 flow (50 mL per min) at 573K (2 ºC per min) for 4 h. After cooling the samples to room temperature, 5%H_2 + 95%N_2 flow was replaced with D_2 flow (50 mL per min) and then the catalyst was heated to 200 ºC (5 ºC per min) during the IR testing. At the beginning and before introducing D_2, the as-prepared reduced Cu-PSNT@m-SiO_2 catalyst (prepared by reducing Cu-PSNT@ m-SiO_2 under H_2 at 300 ºC for 2h) only showed SiO-H with two δ_{OH} bands of hydroxyl groups at 3744 cm^{-1} and 3614 cm^{-1} in FT-IR spectra, and no obvious SiO-D signal was observed[1]. After introducing D_2 and increasing the reaction temperature, SiO-D signal started to appear at 100 ºC, with two δ_{OH} bands at 2622 cm^{-1} and 2758 cm^{-1}, which indicating that the exchange of dissociated H and D at Cu-SiO_2 interface take place[2]. Increasing the reaction temperature would result in much stronger SiO-D signal indicating that the number of SiO-D groups was increased. It's worth to mention that Cu-H cannot be observed because IR peaks of both Cu-H and Cu-D appear at 1852 and 1336 cm^{-1}, near to H_2O (~1640 cm^{-1}), and it is difficult to distinguish these three FT-IR signals [3].

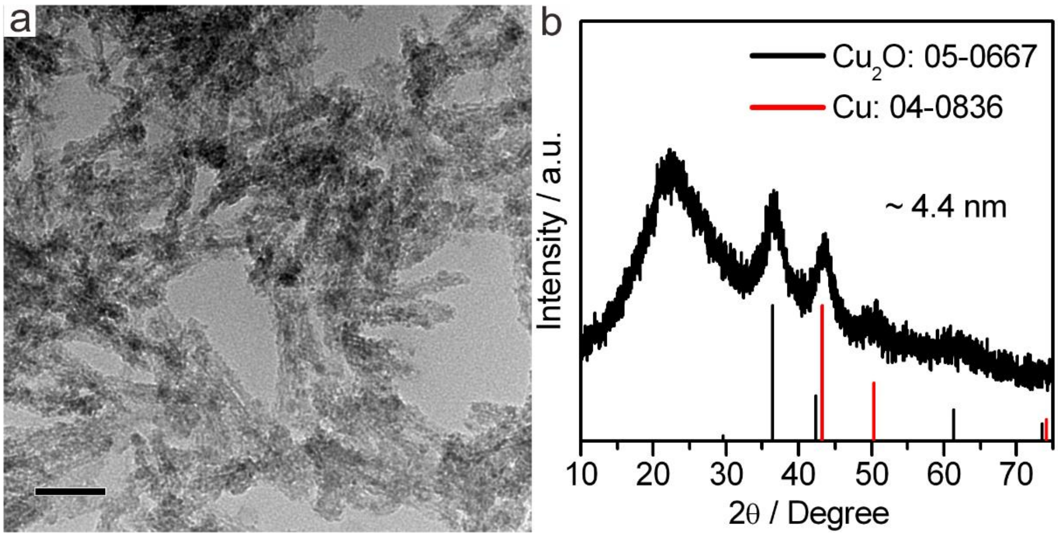

Supplementary Figure 19. Characterizations of Cu-PSNT@m-SiO_2 after catalysis. **a**, **b**, TEM image and X-ray powder diffraction pattern of the reduced Cu-PSNT@m-SiO_2 after LHSV-dependent catalysis test, respectively. Scale bar in a is 50 nm.

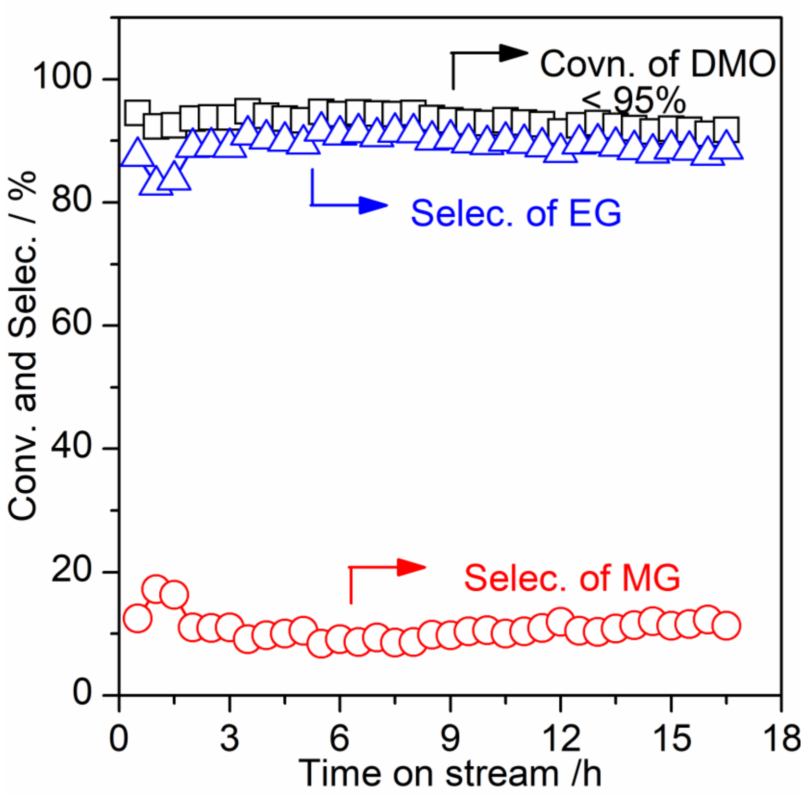

Supplementary Figure 20. Catalytic performance of reduced Cu-PSNT@m-SiO_2 catalyst. Reaction conditions were as follows: H_2 / DMO = 80 mol/mol, P (H_2) = 3.0 MPa, T = 280 ºC, LHSV = 300 h^{-1}.

Supplementary Table 1. The XPS analysis of surface species.

Catalyst	[a] K.E./EV		B.E of Cu $2p_{3/2}$	[b] Cu^+/Cu^0
	Cu^+	Cu^0		
Cu/SiO_2-AE	914.1	917.8	933.0	0.55
Cu-PSNT	914.0	918.1	932.9	0.65
Cu-PSNT@m-SiO_2	914.1	917.8	932.9	0.99

[a] Kinetic energy

[b] Intensity ratio of Cu^+ to Cu^0 from the analysis of Cu XAES spectra

Supplementary Table 2. The TOF of the reduced Cu/SiO_2-AE, the reduced Cu-PSNT and the reduced Cu-PSNT@m-SiO_2 catalysts.

Catalyst	[a] Cu loading (wt %)	[b] Cu dispersion (%)	[c] TOF (h^{-1})
Cu/SiO_2-AE	19.83	54.45	10.21
Cu-PSNT	30.95	35.56	23.08
Cu-PSNT@m-SiO_2	20.47	37.72	40.62
			[d]6254

[a] Determined by ICP-AES.

[b] The copper dispersions were determined by N_2O titration.

[c] The turnover frequencies (TOF) indicated the moles of DMO converted per hour by per mol copper at the catalyst surface. Reaction condition: 473 K, 3.0 MPa, H_2/DMO = 80

[d]Turnover frequency (TOF) was calculated as moles of EG form at LHSV = 4327 h^{-1} in the initial 1h per mole of surface copper calculated form the copper dispersion. Reaction condition: 557 K , 2.5 MPa, H_2/DMO = 200.

Supplementary Table 3. Catalytic performance of Cu-based catalysts for DMO hydrogenation

Catalyst (Cu loading wt %)	LHSV (h^{-1})	P (MPa)	H_2/DMO (mol/mol)	T/K	Conv. (%)	Sel.*/Yield# (%)		Ref.
						EG*	EtOH#	
20%Cu-MCM-41	3.0	2.5	80	473	100	95	/	Ref.[4]
20%Cu-MCM-41	4.5	2.5	80	473	96	39	/	Ref.[4]
Cu/H1S1 (20.0)	1.5	2.5	100	473	100	98	/	Ref.[5]
Cu/H1S1 (20.0)	4.0	2.5	100	473	100	67	/	Ref.[5]
IE-333 (20.4)	0.76	2.5	100	473	100	75	/	Ref.[6]
20Cu/SiO_2 (19.2)	2.5	2.5	200	553	100	/	83.0	Ref.[7]
Cu@CuPSNTs-in (39.3)	2.0	2.5	200	553	98.7	/	91.7	Ref.[8]
CuSi-363 (17.6)	0.5	2.5	50	473	80	42	/	Ref.[9]
1B-Cu-SiO_2 (27.0)	6.0	3.0	80	463	61.9	39.4	/	Ref.[10]
Cu-PSNT@m-SiO_2 (20.5)	3.0	3.0	80	473	99.9	98.4	/	This work
	4.2	3.0	80	473	99.9	98.7	/	
	7.8	3.0	80	473	99.9	96.0	/	
	100	2.5	80	553	99.78	92.3	0.19	
	100	2.5	100	553	98.5	90.6	0.35	
	250	2.5	200	553	95.6	86.2	0.19	

Supplementary References

1 Mawhinney, D. B., Glass, J. A. & Yates, J. T. FTIR Study of the Oxidation of Porous Silicon. *The J. Phys. Chem. B* **101**, 1202-1206, (1997).

2 Gupta, P., Dillon, A., Bracker, A. & George, S. FTIR studies of H2O and D2O decomposition on porous silicon surfaces. *Surf. Sci.* **245**, 360-372, (1991).

3 Ozin, G. A., Mitchell, S. A. & Prieto, J. G. Cu-H2 Photochemistry in the Matrix; ESR, FTIR, UV/VIS Spectroscopic and Kinetic Studies. *Angew. Chem. Int. Ed.* **21**, 380-381, (1982).

4 Ma, X. *et al.* Hydrogenation of dimethyl oxalate to ethylene glycol over mesoporous Cu-MCM-41 catalysts. *AIChE J.* **59**, 2530-2539, (2013).

5 Yin, A. Y., Wen, C. O., Dai, W. L. & Fan, K. N. A. Surface modification of HMS material with silica sol leading to a remarkable enhanced catalytic performance of Cu/SiO_2. *Appl. Surf. Sci.* **257**, 5844-5849, (2011).

6 Yin, A., Guo, X., Fan, K. & Dai, W.-L. Ion-Exchange Temperature Effect on Cu/HMS Catalysts for the Hydrogenation of Dimethyl Oxalate to Ethylene Glycol. *ChemCatChem* **2**, 206-213, (2010).

7 Gong, J. *et al.* Synthesis of Ethanol via Syngas on Cu/SiO_2 Catalysts with Balanced Cu^0-Cu^+ Sites. *J. Am. Chem. Soc.* **134**, 13922-13925, (2012).

8 Yue, H. *et al.* A copper-phyllosilicate core-sheath nanoreactor for carbon–oxygen hydrogenolysis reactions. *Nat. Commun.* **4**, (2013).

9 Chen, L. *et al.* Cu/SiO_2 catalysts prepared by the ammonia-evaporation method: Texture, structure, and catalytic performance in hydrogenation of dimethyl oxalate to ethylene glycol. *J. Catal.* **257**, 172-180, (2008).

10 He, Z., Lin, H., He, P. & Yuan, Y. Effect of boric oxide doping on the stability and activity of a Cu-SiO_2 catalyst for vapor-phase hydrogenation of dimethyl oxalate to ethylene glycol. *J. Catal.* **277**, 54-63, (2011).
